# Pituitary Suppression with Gonadotropin-Releasing Hormone Agonist Prior to Artificial Endometrial Preparation in Frozen–Thawed Embryo Transfer Cycles: A Systematic Review and Meta-Analysis of Different Protocols and Infertile Populations

**DOI:** 10.3390/biomedicines12040760

**Published:** 2024-03-29

**Authors:** Nguyen-Tuong Ho, Dang Khanh Ngan Ho, Xuan Hong Tomai, Nam Nhat Nguyen, Hung Song Nguyen, Yu-Ming Hu, Shu-Huei Kao, Chii-Ruey Tzeng

**Affiliations:** 1 Taipei Fertility Center, Taipei 110, Taiwan or nguyentuonghnt@gmail.com (N.-T.H.); hwu4416@gmail.com (Y.-M.H.); 2 College of Medicine, Taipei Medical University, Taipei 110, Taiwan; 3 IVFMD, My Duc Hospital, Ho Chi Minh City 700000, Vietnam; 4 School of Nutrition and Health Sciences, College of Nutrition, Taipei Medical University, Taipei 110, Taiwan; 5 Office of International Relations, University of Medicine and Pharmacy, Ho Chi Minh City 700000, Vietnam; tomaixuanhong@ump.edu.vn; 6 Division of Infectious Disease, Department of Pediatrics, Pham Ngoc Thach University of Medicine, Ho Chi Minh City 700000, Vietnam; 7 School of Medical Laboratory Science and Biotechnology, College of Medical Science and Technology, Taipei Medical University, Taipei 110, Taiwan; 8 Ph.D. Program in Medical Biotechnology, College of Medical Science and Technology, Taipei Medical University, Taipei 110, Taiwan

**Keywords:** gonadotropin-releasing hormone agonist, pituitary suppression, frozen–thawed embryo transfer, pregnancy outcomes

## Abstract

This study investigates the effect of GnRHa pretreatment on pregnancy outcomes in artificial endometrial preparation for frozen–thawed embryo transfer (AC-FET) cycles. A systematic review of English language studies published before 1 September 2022, was conducted, excluding conference papers and preprints. Forty-one studies involving 43,021 participants were analyzed using meta-analysis, with a sensitivity analysis ensuring result robustness. The study found that GnRHa pretreatment generally improved the clinical pregnancy rate (CPR), implantation rate (IR), and live birth rate (LBR). However, discrepancies existed between randomized controlled trials (RCTs) and observational studies; RCTs showed no significant differences in outcomes for GnRHa-treated cycles. Depot GnRHa protocols outperformed daily regimens in LBR. Extended GnRHa pretreatment (two to five cycles) significantly improved CPR and IR compared to shorter treatment. Women with polycystic ovary syndrome (PCOS) saw substantial benefits from GnRHa pretreatment, including improved CPR and LBR and reduced miscarriage rates. In contrast, no significant benefits were observed in women with regular menstruation. More rigorous research is needed to solidify these findings.

## 1. Introduction

Gonadotropin-releasing hormone agonists (GnRHa) are synthetic versions of the naturally occurring GnRH hormone. They are designed to have a longer half-life by replacing a specific amino acid in the native hormone with a different form, making it resistant to degradation. This results in prolonged receptor occupancy, enhancing its therapeutic effects, such as suppressing spontaneous ovulation during a controlled ovarian hyperstimulation (COH) cycle. Generally, continuous GnRHa administration desensitizes the pituitary gland by causing GnRH receptor downregulation after the initial “flare” response. Although recent wide-spread use of GnRH antagonist protocols ameliorates the importance of GnRHa in IVF/ICSI cycles due to being a more time-consuming treatment with a higher rate of ovarian hyperstimulation syndrome, COH long protocols with GnRHa can still be considered a first-line treatment for patients with advanced age or endometriotic disorders [1,2], as these drugs decrease cancellation rate through the prevention of premature LH surge and luteinization and enhancement of follicular recruitment, allowing the recovery of a larger number of oocytes and improvement in routine patient treatment schedule [3].

Although steroid hormones are important in reproduction, excessive and sustained exposure to sex steroids has been proven to impair endometrial receptivity [4,5]. Additionally, the intrauterine microenvironment, provided mostly by glandular secretions, is crucial for implantation. Certain inflammatory conditions, such as adenomyosis or polycystic ovarian syndrome, induce an aberrant implantation process, reducing the pregnancy rate [6,7]. Pituitary suppression with a GnRH agonist before embryo transfer could suppress the hypothalamic-pituitary-gonadal axis and theoretically create better endometrial-embryonic synchronization and microenvironment for fertilization. In mice, GnRHa ameliorates the adverse impact of adenomyosis on endometrial receptivity by increasing the quality and quantity of pinopodes, as well as the expression of Hoxa10, Hoxa11, Lif, and integrin b3 during the implantation window [8]. An et al. promoted that depot GnRH agonist administration before artificial endometrial preparation improved pregnancy outcomes [9] by regulating the decidualization markers. Another study revealed that GnRHa pretreatment upregulated implantation-related interleukin 6 and 11 in human endometrial stromal cells [10]. In summary, preclinical data on GnRHa and implantation have demonstrated favorable outcomes, implying that the use of GnRHa prior to transfer could be a feasible option for infertile women. Nevertheless, the translation of a medication from preclinical to clinical settings has not always been successfully achieved. The determination of an optimal treatment modality should be predicated upon the clinical context of the individual patient. It is imperative to acknowledge that there is no universally superior treatment applicable to all patients, as each individual’s physiological constitution presents a distinct clinical scenario necessitating a bespoke approach. As the GnRHa-pretreated FET protocol has been considered significantly costly in money and time compared to a conventional approach [11], the decision to use this regimen should be based on the unique characteristics of the patients rather than being applied routinely. Additionally, there have been ongoing discussions regarding the optimal type and duration of GnRHa used in assisted reproductive technology (ART) in terms of cost-effectiveness, patient convenience, and efficacy. While depot GnRHa may require a single high dose for pituitary suppression, the daily low-dose GnRHa protocol involves a lower total dosage but a higher number of injections [12]. In certain cases, such as adenomyosis, longer pituitary suppression using GnRHa may contribute to better treatment outcomes [13], but it also carries an increased risk of side effects [14]. Therefore, it is necessary to further clarify the most suitable approach for the use of GnRHa prior to frozen embryo transfer (FET) in order to achieve the highest success rate.

Through meta-analysis, this study systematically assessed clinical studies focused on the effects of GnRHa treatment before FET with artificial cycles (AC-FET). With more updated and relevant data available, we compared the effectiveness of AC-FET cycles with and without GnRHa pretreatment and the difference in pregnancy outcome between different GnRHa protocols and treatment durations among infertile women suffering a variety of infertility etiologies.

## 2. Methods

### 2.1. Search Strategy and Study Selection

This systematic review and meta-analysis followed the Preferred Reporting Items for Systematic Reviews and Meta-Analysis checklist (PRISMA 2020, Supplementary Table S1). The search was conducted in four primary electronic databases on 19 January 2022: PubMed, EMBASE, Google Scholar, and the Cochrane Library. After screening full texts, we updated our search on 30 August 2022 to obtain more related articles. A manual search was also performed by screening the references of the included and related studies suggested by PubMed and Google Scholar, as listed on the first page of Supplementary Table S2. The suggested keywords were: “(pituitary suppression OR GNRHa OR gonadotropin-releasing hormone agonist) AND (FET OR frozen–thawed embryo transfer) AND (Artificial cycle OR HRT OR Hormonal replacement therapy OR HRC OR Hormonal replacement cycle)”. Exclusion criteria were studies with unreliable clinical data, analyses with overlapping data sets, full-text articles not available, non-English articles, book chapters, abstract-only articles, letters, editorials, correspondence, theses, conference papers, reviews, animal studies, case reports, and case series. Additional articles were also retrieved through a manual search. We used Endnote (version 20; Clarivate. Philadelphia, PA, USA) to manage the studies found.

### 2.2. Population, Intervention, Comparison, Outcomes, and Study Design (PICOS)

Participants included patients indicated for frozen–thawed embryo transfer with artificial endometrial preparation, regardless of infertile etiologies. We conducted comparative meta-analyses to assess the efficacy of pituitary suppression prior to hormonal therapy initiation in FET patients compared to a non-pretreated control group in terms of pregnancy outcomes. Pituitary suppression was managed using GnRHa in short-acting (daily) or long-acting (depot) protocols. The duration of GnRHa administration (number of depot doses or number of treated cycles) was also recorded and analyzed.

The primary outcome was clinical pregnancy rate (CPR), defined as the presence of at least one intrauterine gestational sac (yolk-sac) with or without fetal heart activity under vaginal ultrasound examination.

Secondary outcomes were implantation rate (IR), miscarriage rate (MR), and live birth rate (LBR). Implantation rate was the ratio between the number of sacs observed via ultrasound and embryos transferred. Miscarriage was the loss of one or more intrauterine non-viable fetuses. A live birth was defined as the delivery of one or more fetuses which are viable.

### 2.3. Systematic Review Protocol and Registration

We registered the protocol in the PROSPERO International Prospective Register of Systematic Reviews. The registration number is CRD42022299259.

### 2.4. Data Extraction

In an effort to eliminate potential bias, the search was conducted by three separate researchers. The data collected from the studies included the study design, patient demographics, clinical characteristics, and measured outcomes, which were then compared and evaluated among the three individuals. In instances of disagreement, a discussion and voting process were utilized to arrive at a consensus.

Study quality and risk of bias were evaluated by two independent researchers using the Effective Public Health Practice Project (EPHPP) Quality Assessment Tool for Quantitative Studies [15]. The EPHPP is composed of eight domains, which include analysis, withdrawals and dropouts, data collection practices, selection bias, intervention integrity, blinding, and confounders. Two domains (analysis and intervention integrity) are descriptive and were not used for global rating. For the remaining 6 domains, each domain is rated as weak (1 point), moderate (2 points), or strong (3 points) and the overall quality of a trial is rated as low, moderate, or strong.

### 2.5. Data Analysis

The study’s effect and mean weight were visualized using forest plots and odds ratios (OR) with 95% confidence intervals (95% CIs). The *I*^2^ statistic was employed to assess heterogeneity. According to the Cochrane Handbook for Systematic Reviews of Interventions, an *I*^2^ value of 0 indicates no observed heterogeneity, *I*^2^ values from 50–75% represent moderate heterogeneity, and *I*^2^ values > 75% indicate high heterogeneity. A random-effects model is used when there is heterogeneity between studies, as confirmed by a Cochran’s Q test *p*-value of 0.1 or an *I*^2^ of more than 50%. A fixed-effects model was preferred in all other cases. Subgroup analysis was used to investigate sources of heterogeneity. The effectiveness of GnRHa on each specific infertile population was reported in meta-analyses of subset data. Sensitive analysis was performed with the presence of publication bias investigated by Egger’s asymmetric test. We analyzed data using R software (version 4.2.2; R Foundation for Statistical Computing; Vienna, Austria), with a two-sided *p*-value of <0.05 considered statistical significance.

## 3. Results

### 3.1. Literature Search and Study Selection

**Figure 1:**
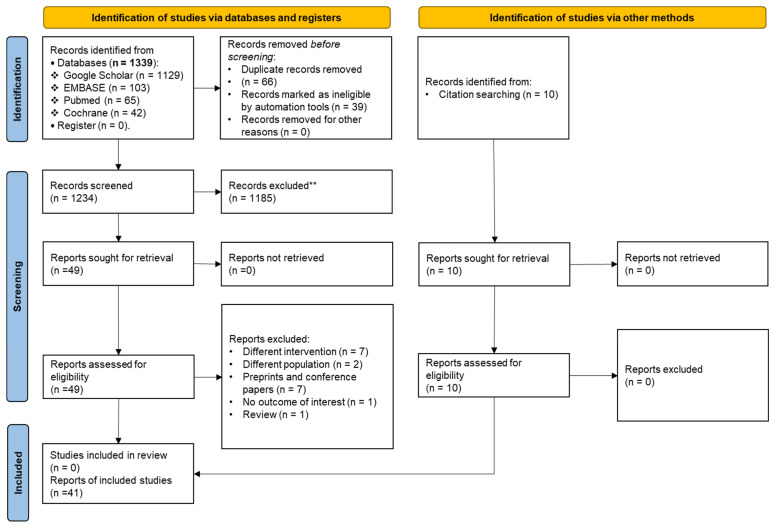
PRISMA flowchart. **: Records that were irrelevant to the research question.

A total of 1349 articles were identified from the databases through a systematic search in combination with a manual search of relevant citations (Figure 1). Next, articles remaining after deduplication were screened for their titles and abstracts. Of these articles, 1290 were excluded due to duplication (*n* = 66), irrelevancy as detected by automation tools (*n* = 39), and manual screening (*n* = 1185). Fifty-nine papers remained for the eligibility assessment. Another 18 publications were further excluded because they did not include the outcome of interest (*n* = 1); reported pituitary suppression prior to IVF/ICSI cycles (*n* = 2) or GnRHa administration for luteal support (*n* = 7); or were preprints (*n* = 7), conference papers (*n* = 5), and a review (*n* = 1). Finally, 41 studies met our inclusion criteria for a systematic review (Table 1) and were pooled in the meta-analyses.

### 3.2. Study and Participant Characteristics

**Figure 2:**
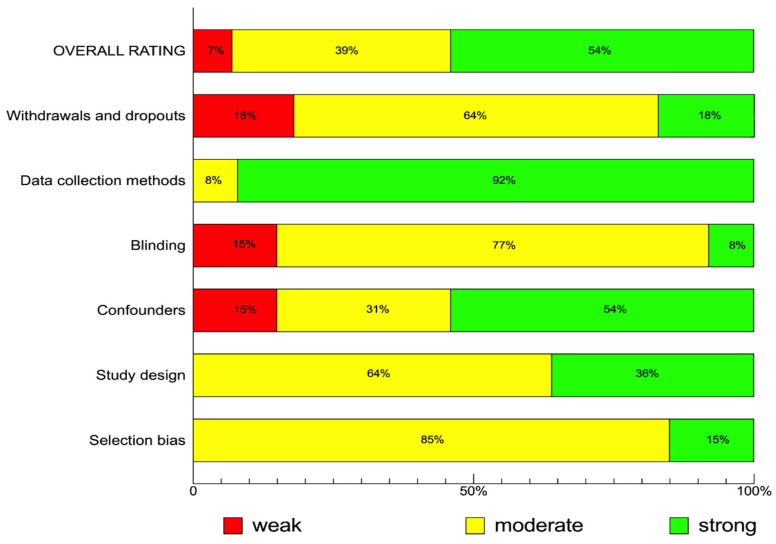
Summary of study quality assessment using the Effective Public Health Practice Project (EPHPP) quality assessment tool (*n* = 41 studies)—Data shown as percentage of number of studies.

A total of 43,021 participants were recruited in the studies [9,10,11,16,17,18,19,20,21,22,23,24,25,26,27,28,29,30,31,32,33,34,35,36,37,38,39,40,41,42,43,44,45,46,47,48,49,50,51,52,53]. The final systematic review comprised fourteen randomized controlled trials and twenty-seven observational studies, among which were two non-randomized prospective studies, two case–control studies, nineteen retrospective cohort studies, and four retrospective cohort studies matched using propensity score matching (PSM). Xia et al. (2022) reported the effectiveness of GnRHa administration prior to FET in three cohorts of women with no previous implantation failure, one previous implantation failure, or multiple previous implantation failures [48]. The first two cohorts were analyzed via the PSM approach, while the latter was reported without matching. According to the difference in analysis method, these cohorts were analyzed separately. The EPHPP assessment results revealed that most studies were rated as having adequate quality (Figure 2). The inclusion and exclusion of each study are listed in Supplementary Table S3.

Egger’s test revealed publication biases in the overall CPR, LBR, and MR, as shown in Supplementary Table S4. In order to explore heterogeneity, we conducted a sensitivity analysis using Baujat’s method and utilized Baujat plots to identify sources of heterogeneity (Supplementary Figures S1–S3) [54]. The exclusion of outliers via this method did not alter the final results, as evidenced by Supplementary Table S4. No publication bias was observed in other outcomes or subgroup analyses.

### 3.3. Main Findings

#### 3.3.1. FET Outcomes between Cycles with and without GnRHa Pretreatment

**Figure 3:**
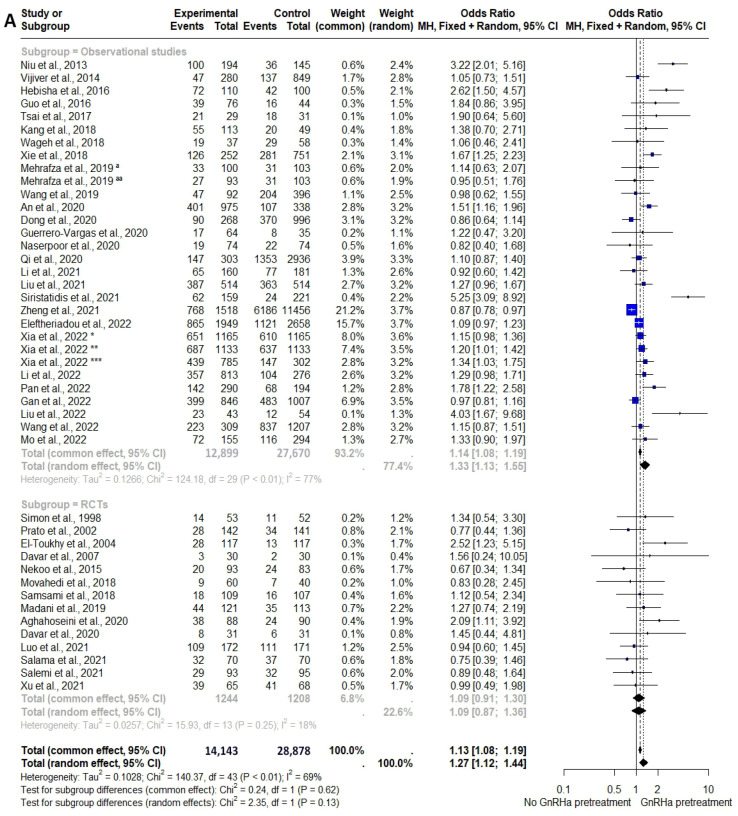

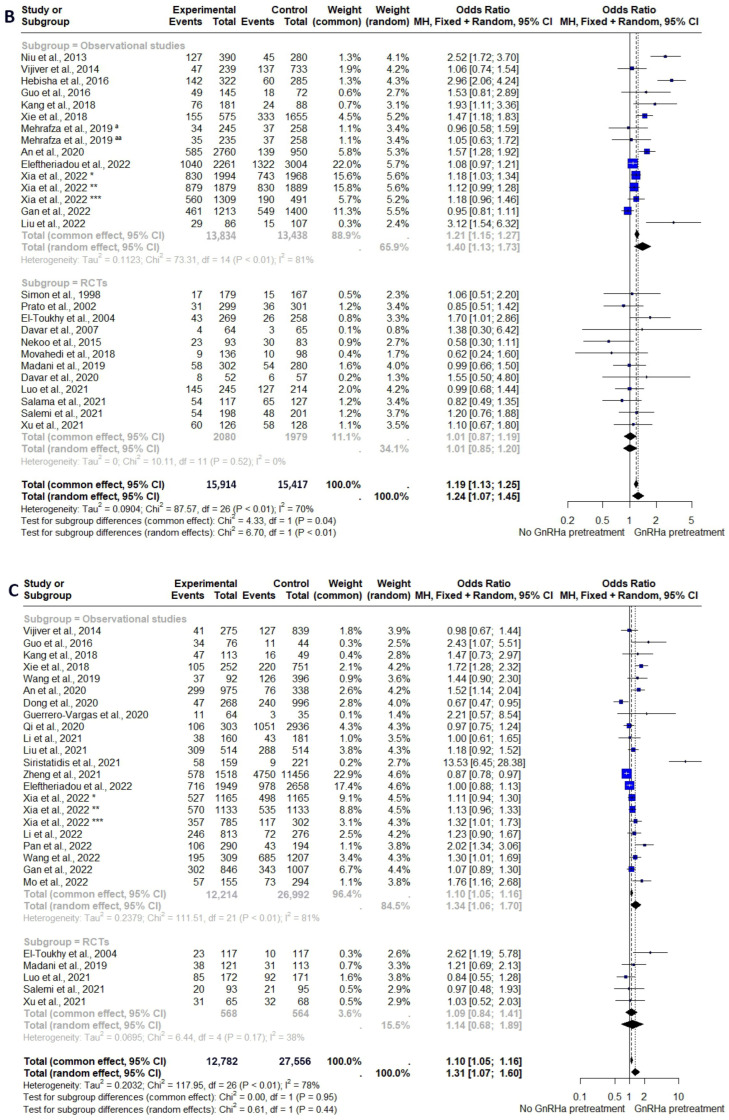

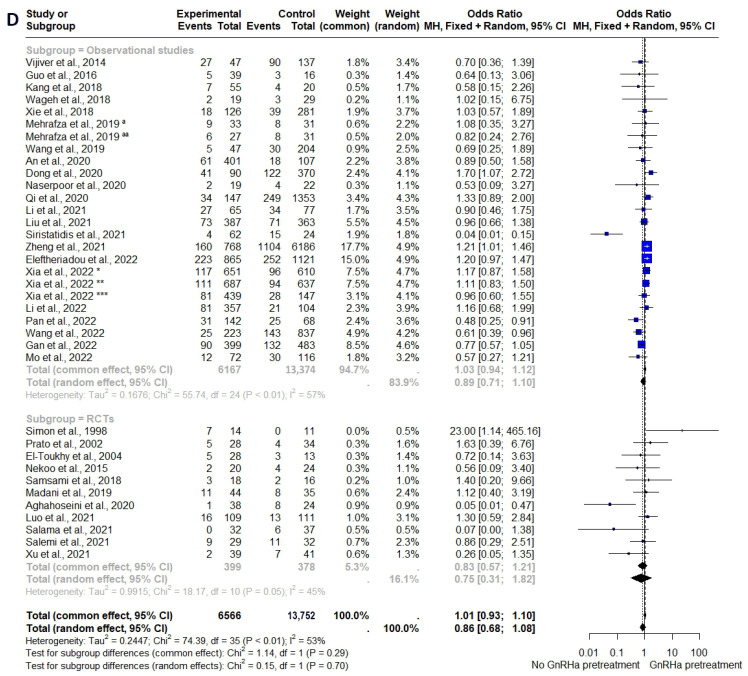
Forest plots of meta-analysis for pregnancy outcomes following AC-FET cycles with and without GnRHa pretreatment [9,10,11,16,17,18,19,20,21,22,23,24,25,26,27,28,29,30,31,32,33,34,35,36,37,38,39,40,41,42,43,44,45,46,47,48,49,50,51,52,53]. (**A**) Clinical Pregnancy Rate. (**B**) Implantation Rate. (**C**) Live Birth Rate. (**D**) Miscarriage Rate. *, **, and ***: three populations of infertile women were reported in the same study with matched or non-matched designs. ^a^ and ^aa^: two different protocols were applied in the same study. RCTs: randomized controlled trials.

Overall, pituitary suppression with GnRHa significantly improved the CPR (OR = 1.27, 95% CI: 1.12–1.44, *I*^2^ = 69.4%, *p* < 0.001), IR (OR = 1.24, 95% CI: 1.07–1.45, *I*^2^ = 70.3%, *p* = 0.006), and LBR (OR = 1.31, 95% CI: 1.07–1.60, *I*^2^ = 78.0%, *p* = 0.01) except for MR (OR = 0.86, 95% CI: 0.68–1.08, *I*^2^ = 53.0%, *p* = 0.38) (Figure 3).

However, there were discrepancies in subgroup analysis for the study design. While subgroup analysis on observational studies demonstrated favorable outcomes, subgroup analysis on RCTs promoted non-significantly different chances of pregnancy among GnRHa-pretreated FET cycles in comparison to conventional AC-FETs (Table 2 (A) and Figure 3). Albeit insignificant, analysis of RCTs still demonstrated a slightly better LBR among women receiving GnRHa prior to embryo transfer. The limited number of participants in RCTs included in this analysis (1244 cycles with GnRHa and 1208 controls) could contribute to the results.

**Table 1:** Characteristics of the studies in the systematic review and meta-analysis.

Author	Country	Research Design	Number of Participants (Case/Control)	Diagnosis of Participants	Drug Use	Artificial Endometrial Preparation Protocol	Protocol
Simon A. (1998) [16]	Israel	RCT	53/53	Mixed	Triptorelin pamoate	Step-up	One dose of depot GnRHa 3.75 mg IM at preceding early follicular phase (irregular cycle) or mid-luteal phase (regular cycle)
Prato L. D. (2002) [17]	Italy	RCT	146/150	Tubal, idiopathic, or male factors	Triptorelin pamoate	Step-up	One dose of depot GnRHa 3.75 mg IM at preceding mid-luteal phase
El-Toukhy T. (2004) [18]	United Kingdom	RCT	117/117	Mixed	Bureselin acetate	Fixed-dose	GnRHa 400 mcg nasally every day from preceding mid-luteal phase to the day before P4 administration
Davar R. (2007) [19]	Iran	RCT	30/30	Mixed	Bureselin acetate	Step-up	Daily GnRHa 0.5 mg SC daily from preceding mid-luteal phase to the day before P4 administration
Niu Z. (2013) [20]	China	Retrospective cohort study	194/145	Adenomyosis	Leuproreline acetate	Step-up	Two doses of depot GnRHa: 1st dose: 3.75 mg IM and 2nd dose: 1.875 mg IM at 2 consecutive early follicular phase
Vijiver A. (2014) [21]	Belgium	Retrospective cohort study	280/849	Mixed	Bureselin acetate	Step-up	GnRHa 600 mcg nasally every day from preceding mid-luteal phase to the day before P4 administration
Nekoo E. A. (2015) [22]	Iran	RCT	93/83	Male factor	Triptorelin pamoate	Step-up	One dose of depot GnRHa 3.75 mg IM at preceding mid-luteal phase
Hebisha S. (2016) [24]	Egypt	Prospective cohort study	110/100	Mixed	Triptorelin acetate	Fixed-dose	Daily GnRHa—0.1 mg SC from preceding mid-luteal phase—0.05 mg SC from E2 administration day to day before P4 administration
Guo S. (2016) [23]	China	Retrospective cohort study	76/44	Adenomyosis	Triptorelin acetate	NR	Depot GnRHa—one dose 3.75 mg IM—early follicular phase
Tsai H. W. (2017) [25]	Taiwan	Retrospective cohort study	29/31	PCOS	Leuprolide acetate	Fixed-dose	Depot GnRHa—two dose 3.75 mg IM
Kang J. (2018) [26]	Korea	Retrospective cohort study	113/49	Mixed with exclusion of PCOS	Buserelin acetate	Step-up	Daily GnRHa—0.1 mg SC from preceding mid-luteal phase for 14 days
Movahedi S. (2018) [27]	Iran	RCT	60/40	Mixed with exclusion of endometriosis	Buserelin acetate	Step-up	Daily GnRHa—0.5 mg SC from preceding mid-luteal phase
Samsami A. (2018) [28]	Iran	RCT	109/107	Mixed	Buserelin acetate	Step-up	Daily GnRHa—0.5 mg SC from preceding mid-luteal phase, 0.3 mg SC from E2 administration day
Wageh A. (2018) [29]	Egypt	Retrospective cohort study	37/58	PCOS	Triptorelin acetate	Step-up	Daily GnRHa—0.1 mg SC from preceding mid-luteal phase
Xie D. (2018) [30]	China	Retrospective cohort study	252/751	Mixed	Leuprorelin acetate	Step-up	Depot GnRHa—one or two dose (per 4 week) 3.75 mg IM—early follicular phase
Madani T. (2019) [31]	Iran	RCT	121/113	Mixed	Buserelin acetate	Step-up	Daily GnRHa—0.5 mg SC from preceding mid-luteal phase
Mehrafza M. (2019) [32]	Iran	Retrospective cohort study	193/103	Mixed	Bureselin acetate/ Triptorelin pamoate	Step-up	Daily GnRHa—0.3 mg SC from preceding mid-luteal phase—0.2 mg SC from E2 administration to day 6 or depot GnRHa—one dose 1.875 mg IM—mid-luteal phase
Wang Z. (2019) [33]	China	Retrospective cohort study	92/396	Endometrial polyp	Bureselin acetate	Step-up	Depot GnRHa—one dose 0.8-3.75 mg IM—mid-luteal phase
Aghahoseini M. (2020) [34]	Iran	RCT	88/90	PCOS	Triptorelin acetate	Step-up	Depot GnRHa 3.75 mg—two doses with an interval of 4 weeks, beginning at 8 weeks before estradiol administration
An J. (2020) [9]	China	Retrospective cohort study	975/338	Mixed with exclusion of endometriosis	Leuprolide acetate	Fixed-dose	Depot GnRHa 1.875 mg—mid-luteal phase—one to three doses for each three weeks
Davar R. (2020) [35]	Iran	RCT	34/33	RIF	Triptorelin acetate	Step-up	Daily GnRHa—0.1 mg SC from preceding mid-luteal phase—0.05 mg SC from E2 administration day to day before P4 administration
Dong M. (2020) [36]	China	Retrospective cohort study	268/996	Elderly patients	NR	Step-up	Depot GnRHa—one dose 3.75 mg IM—early follicular phase
Guerrero-Vargas J. J. (2020) [37]	Spain	Retrospective cohort study	64/35	Mixed	Leuprolide acetate/triptorelin acetate	Step-up	Daily GnRHa—1 mg SC (Leuprolide acetate) or 0.1 mg (triptorelin acetate) from preceding mid-luteal phase, then reduce by half if pituitary suppression achieved
Naserpoor L. (2020) [38]	Iran	Retrospective case–control study	74/74	Mixed	Buserelin acetate	Step-up	0.5 mg/day initiated from the 19th day of the previous menstrual cycle, then reduce by half at E2 initiation
Qi Q. (2020) [39]	China	Retrospective cohort study	303/2936	Mixed	Leuprorelin acetate/ Triptorelin acetate	Fixed-dose	Depot GnRHa—one dose 3.75 mg IM at preceding early follicular phase
Li M. (2021) [40]	China	Retrospective cohort study	160/181	Adenomyosis	Triptorelin/ Leuproreline	Step-up	Depot GnRHa ≥ one dose 3.75 mg IM at early follicular phase each month
Liu X. (2021) [41]	China	PSM retrospective cohort study	514/514	PCOS	Triptorelin acetate	Step-up	Depot GnRHa—1 dose 3.75 mg IM—early follicular phase
Luo L. (2021) [11]	China	RCT	172/171	PCOS	Triptorelin acetate	Step-up	Depot GnRHa—1 dose 1 mg IM—early follicular phase
Salama K. M. (2021) [42]	Egypt	RCT	70/70	Mixed	Triptoreline acetate	Step-up	One dose depot GnRHa 3.75 mg—mid-luteal phase
Salemi S. (2021) [43]	Iran	RCT	106/106	PCOS	Bureselin acetate	Step-up	Daily GnRHa—0.5 mg SC from preceding mid-luteal phase for 14 days
Siristatidis C. (2021) [44]	Greece	Retrospective cohort study	159/221	Normal ovulatory women without PCOS	NR	Step-up	Daily GnRHa—dose NR
Xu J. (2021) [45]	China	RCT	65/68	Mixed with exclusion of endometriosis and PCOS	Triptorelin acetate	Fixed-dose	Depot GnRHa—1 dose 3.75 mg IM—early follicular phase
Zheng Q. Z. (2021) [46]	China	Retrospective cohort study	1518/11,456	Mixed	Leuproreline acetate	Step-up	Depot GnRHa—one dose 3.75 mg IM—mid-luteal phase
Eleftheriadou A. (2022) [47]	United Kingdom	Non-randomized prospective cohort study	1949/2658	Mixed	Buserelin acetate	Step-up	Daily GnRHa—0.5 mg SC from preceding mid-luteal phase until P4 commencement
Xia L. (2022) * [48]	China	PSM retrospective cohort study	1165/1165	Women without previous FET failure	Triptorelin embonate	Step-up	Depot GnRHa—one dose 3.75 mg IM—early follicular phase
Xia L. (2022) ** [48]	China	PSM retrospective cohort study	1133/1133	Women with one previous FET failure	Triptorelin embonate	Step-up	Depot GnRHa—one dose 3.75 mg IM—early follicular phase
Xia L. (2022) *** [48]	China	Retrospective cohort study	785/302	Women with more than one FET failure	Triptorelin embonate	Step-up	Depot GnRHa—one dose 3.75 mg IM—early follicular phase
Li L. (2022) [10]	China	Retrospective cohort study	853/290	Women with ovulation and regular cycle	Leuprorelin acetate	Fixed-dose	Depot GnRHa 1.875 mg at mid-luteal phase for 3-5 cycles consecutively
Pan D. (2022) [49]	China	Retrospective cohort study	290/194	Older patients >35 yrs with RIF and without adenomyosis and endometriosis	Triptorelin acetate	Step-up	Depot GnRHa—one dose 3.75 mg IM—early follicular phase
Wang Y. (2022) [50]	China	PSM retrospective cohort study	309/1207	PCOS	Triptorelin embonate	Step-up	Depot GnRHa—one dose 3.75 mg IM—early follicular phase
Gan R. X. (2022) [51]	China	Retrospective cohort study	846/1007	Women with history of cesarean scar(s)	Triptorelin acetate	Fixed-dose and Step-up	Depot GnRHa—one dose 1.875 mg IM—early follicular phase
Mo M. (2022) [52]	China	PSM retrospective cohort study	155/294	women with history of intrauterine adhesion	Leuprorelin acetate	Step-up	Depot GnRHa—one dose 3.75 mg IM—mid-luteal phase
Liu Y. (2022) [53]	China	Retrospective case–control study	43/54	Women with persistent thin endometrium	Leuprorelin acetate	Step-up	Depot GnRHa—1st dose: 1.5 mg IM At early follicular phase and 28 days later 2nd dose: 1.5 mg IM (14 days before E2 initiation)

*, **, and ***: three populations of infertile women were reported in the same study with matched or non-matched designs. PCOS: Polycystic Ovarian Syndrome; PSM: Propensity Score Matching; GnRHa: Gonadotropin releasing hormone agonist. NR: Non-Reported. P4: progesterone; E2: Estradiol; IM: Intramuscular; SC: Subcutaneous RCT: Randomized Controlled Trial; RIF: Repeated Implantation Failure.

#### 3.3.2. Subgroup Meta-Analysis of Different Down-Regulation Protocols and Treatment Durations of Pituitary Suppression with GnRHa Prior to Artificial FET Cycles

All subgroup meta-analyses in this study were conducted using a random-effects model, chosen in response to the significant heterogeneity observed within the overall study population. The short-acting regimen (daily protocol) was associated with higher CPR (OR = 1.11, 95% CI: 1.02–1.21, *I*^2^ =0.0%, *p* = 0.02) compared with the control. The differences in IR (OR = 1.17, 95% CI: 0.96–1.42, *I*^2^ = 1.7%, *p* = 0.08), LBR (OR = 1.14, 95% CI: 0.86–1.52, *I*^2^ = 26.8%, *p* = 0.18) and MR (OR = 1.10, 95% CI: 0.95–1.28, *I*^2^ = 0.0%, *p* = 0.16), however, were not considerably different. On the other hand, pituitary suppression with long-acting GnRHa (depot protocol) improved CPR (OR = 1.25, 95% CI: 1.08–1.44, *I*^2^ = 72.1%, *p* = 0.004), IR (OR = 1.28, 95% CI: 1.02–1.59, *I*^2^ = 78.9%, *p* = 0.03), and also LBR (OR = 1.19, 95% CI: 1.04–1.37, *I*^2^ = 72.1%, *p* = 0.02), accompanied by insignificant changes in MR (OR = 0.93, 95% CI: 0.75–1.15, *I*^2^ = 48.2%, *p* = 0.48). Comparing the two protocols, pregnancy outcomes after FET seemed to be slightly improved with depot GnRHa administration. However, their differences were not significant (Table 2 (B) and Supplementary Figure S4).

Women who were pretreated within one cycle prior to FET had better CPR (OR = 1.17, 95% CI: 1.04–1.32, *I*^2^ = 61.3%, *p* = 0.01), IR (OR = 1.16, 95% CI: 1.02–1.32, *I*^2^ = 56.7%, *p* = 0.03), and LBR (OR = 1.28, 95% CI: 1.03–1.60, *I*^2^ = 77.8%, *p* = 0.03). After pituitary suppression, MR did not differ from the non-pretreated group (OR = 0.87, 95% CI: 0.69–1.09, *I*^2^ = 53.7%, *p* = 0.21). Moreover, women who were pretreated more than one cycle prior to FET demonstrated, to a greater extent, improvements in CPR (OR = 2.00, 95% CI: 1.29–3.10, *I*^2^ = 66.4%, *p* = 0.01), IR (OR = 2.07, 95% CI: 0.97–4.43, *I*^2^ = 64.4%, *p* = 0.05), while LBR (OR = 1.42, 95% CI: 0.24–8.39, *I*^2^ = 37.9%, *p* = 0.24) and the rate of miscarriage (OR = 0.50, 95% CI: 0.01–24.11, *I*^2^ = 72.6%, *p* = 0.52) were not significantly different compared with the control. In summary, compared to short-term suppression, prolonged GnRHa administration resulted in better CPR and IR (*p* < 0.05), though no significant differences were found in LBR or MR (Table 2 (C) and Supplementary Figure S5).

#### 3.3.3. Subset Meta-Analyses of Specific Etiologies among Infertile Women Undergoing FET with Hormonal Endometrial Preparation

**Figure 4:**
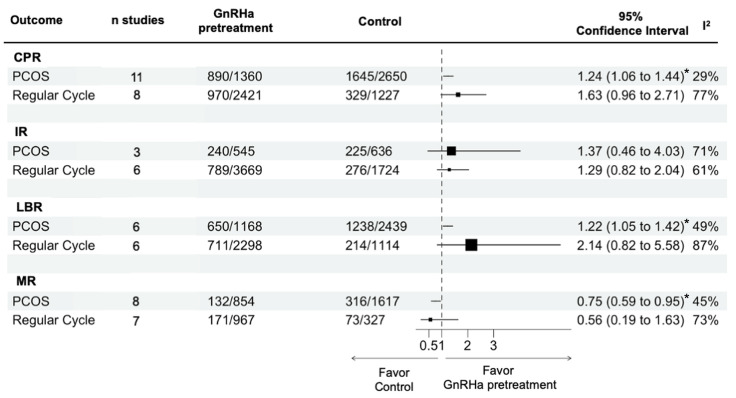
Subset meta-analyses on specific populations: women with PCOS and women with regular menstruation and no ovulation disorders. CPR: clinical pregnancy rate, IR: implantation rate, LBR: live birth rate, MR: miscarriage rate. *: statistically significant.

In PCOS patients, GnRHa pretreatment promoted higher CPR (OR = 1.24, 95% CI: 1.06–1.44, *I*^2^ = 29.1%, *p* = 0.006) and LBR (OR = 1.22, 95% CI: 1.05–1.42, *I*^2^ = 48.9%, *p* = 0.01), accompanied by a lower MR (OR = 0.75, 95% CI: 0.59–0.95, *I*^2^ = 44.9%, *p* = 0.02) (Figure 4 and Supplementary Figure S6). However, IR (OR = 1.37, 95% CI: 0.46–4.03, *I*^2^ = 71.4%, *p* = 0.35) did not considerably differ from the non-pretreated women.

On the other hand, subset meta-analysis of normal ovulatory women with regular menstruation as an inclusion criterion demonstrated high heterogeneity with no significant improvements in pregnancy outcomes, including CPR (OR = 1.63, 95% CI: 0.98–2.71, *I*^2^ = 76.7%, *p* = 0.06), IR (OR = 1.29, 95% CI: 0.82–2.04, *I*^2^ = 60.7%, *p* = 0.21), LBR (OR = 2.14, 95% CI: 0.82–5.58, *I*^2^ = 86.6%, *p* = 0.10) and MR (OR = 0.56, 95% CI: 0.19–1.63, *I*^2^ = 73.3%, *p* = 0.23) among those pretreated with GnRHa (Figure 4 and Supplementary Figure S7).

## 4. Discussion

Successful implantation must be initiated by interaction between a competent embryo and a receptive endometrium. Alongside efforts to improve embryo quality and euploidy rate, sufficient endometrial preparation and synchronization are also essential for successful implantation. Although certain previous studies have compared different endometrial preparation protocols, the optimal strategy for embryo transfer remains conflicting. In this work, all included studies employed an artificial cycle protocol for endometrial preparation. Some studies employed a fixed-dose approach [10,18,24,25,39,45], while most authors opted for a step-up regimen. Only the study conducted by Gan et al. used both regimens [51]. Typically, the duration of endothelial preparation with estrogen does not exceed 21 days, and the endometrial thickness must surpass 7 mm prior to embryo transfer to prevent cycle cancellation. The sole variable differentiating the intervention group from the control cohort was the application of GnRHa for pituitary downregulation. Nonetheless, there has been a consensus that pregnancy outcomes were not significantly different between the fixed-dose and step-up regimens utilized for AC-FET [55]. According to the above-mentioned facts, we posit that the AC regimen of endometrial preparation does not exert a significant impact on the treatment outcomes. Thus, this report exclusively focused on evaluating the efficacy of pituitary suppression with GnRHa on the outcomes of AC-FET cycles. We additionally included subgroup analyses of specific infertile populations without evaluating the AC regimen type utilized.

GnRHa, which is widely recognized for its ability to suppress the pituitary gland and exhibit anti-inflammatory effects, has become prevalent in the realm of assisted reproductive technologies. In insemination cycles, this GnRH analog can be used in conjunction with fertility drugs to regulate the menstrual cycle and synchronize ovulation in order to optimize the timing of IUI. During COH, sustained GnRHa administration causes refractoriness of the pituitary, avoiding a premature LH surge, while the high-dose bolus of this medication triggers the final maturation of oocytes [56]. An appropriate dose of GnRHa after embryo transfer could retain its stimulatory effect to preserve LH production, as has been recently postulated in a meta-analysis of the efficacy of GnRHa in luteal-phase support during both fresh and frozen cycles [57]. Some in vitro and in vivo studies have enlightened the mechanisms behind how GnRH agonists improve endometrial receptivity and enhance embryo implantation [8,58]. However, the influence of GnRHa on the uterine endometrium and implantation process remains a subject of ongoing debate, with no consensus having been reached thus far. This systematic review and meta-analysis demonstrates that pituitary suppression using GnRHa prior to artificial embryo transfer cycles significantly enhanced pregnancy outcomes in women undergoing artificial FET cycles.

However, there were discrepancies between subgroup analyses of RCTs and observational cohort studies. Meta-analyses on implantation and pregnancy outcomes failed to demonstrate any significant differences (Table 2 (A) and Figure 3). Nevertheless, the subgroup analysis incorporated randomized controlled trials (RCTs) that had inherent limitations in terms of sample size and study quality. Consequently, the evidence derived from these RCTs did not possess sufficient strength to support a definitive conclusion.

GnRHa can be administered in short-acting form as daily low-dose shots or through a single long-acting depot injection. The utilization of depot GnRHa during COH results in a more robust suppression effect, necessitating a higher dose of gonadotropins and an extended period of administration. This may lead to an increase in overall treatment cost as compared with daily low-doses of GnRHa [59]. On the other hand, the depot GnRH-a protocol appeared to offer a significantly higher LBR in normogonadotropic women without discernible differences in luteal function or offspring health, as recently reported by Zhang et al. in a large-scale matched cohort study [60]. The eutopic expression levels of endometrial receptivity markers, such as HOXA10, MEIS1, and LIF, were significantly greater with the depot GnRHa protocol compared to GnRH antagonist or long GnRHa protocols in fresh embryo transfer cycles [61]. In the context of endometrial preparation for FET, daily injections for the GnRHa pretreatment protocol require more visits and injections, thereby potentially increasing the cost of treatment. In this meta-analysis, we documented the beneficial effects of both protocols on pregnancy outcomes (Table 2 (B) and Supplementary Figure S4). Notably, GnRHa depot had an impact on live birth outcomes (OR = 1.19, 95% CI: 1.04–1.37, *I*^2^ = 72.1%, *p* = 0.02), whereas no significant improvement was found in women pretreated with a daily GnRHa regimen (OR = 1.14, 95% CI: 0.86–1.52, *I*^2^ = 26.8%, *p* = 0.18). The result postulates that the use of depot GnRHa could be a superior option for pituitary down-regulation prior to FET cycles compared to daily low-dose administration in terms of cost efficiency, patient convenience, and treatment efficacy.

The choice between short-term or long-term down-regulation with GnRHa in assisted reproductive technology (ART) procedures has been a subject of ongoing debate among reproductive endocrinologists. Sustained GnRHa administration has been specifically considered for women who possess particular medical conditions. GnRHa taken for 3–4 months before fibroid surgery can decrease the size of fibroids and the volume of the uterus, as well as address pre-operative iron deficiency anemia and minimize blood loss during myomectomy or hysterectomy [62]. Conservation treatment for adenomyosis or fibroid has also been considered with long-term GnRH analogues [63], while a post-operative approach with this protocol could reduce the risk of endometriosis recurrence [64]. Longer GnRHa treatment (≥3 months) ameliorates the inflammatory microenvironment [65], thus improving the quality and quantity of retrieved oocytes in IVF cycles among women with endometriosis [66]. We reported herein better pregnancy outcomes after FET with prolonged pituitary suppression (Table 2 (C) and Supplementary Figure S5) when compared with those undergoing GnRHa pretreatment within one cycle. It is essential to emphasize that the therapeutic benefits of GnRHa are intertwined with its associated consequences [67]. Temporary symptoms such as hot flashes, fatigue, and loss of libido typically subside shortly after discontinuing GnRHa. Other detrimental consequences, such as osteoporosis or gynecomastia, usually persist longer but have been suggested to occur only with extremely extended usage of GnRH analogs [68]. In this meta-analysis, only a limited number of studies were found that utilized GnRHa in a multi-cycle manner. Those studies that reported GnRH pretreatment for more than one month exclusively used the depot form and limited the number of depot GnRHa doses to less than six, thus reducing the risk of detrimental side effects. Due to insufficient data, we were unable to compare the effectiveness of different treatment durations on the pregnancy outcomes of FET cycles. Nevertheless, the findings herein support the use of GnRHa for more than one cycle but not exceeding six months. Since the evidence on this protocol remains limited, routine application to all women could non-beneficially increase the time and cost of treatment. More rigorous and well-designed studies are necessary to determine the most effective pituitary suppression protocol before embryo transfer.

In women suffering PCOS, endometrial receptivity has been postulated to be affected via several mechanisms: (1) sustained androgenic exposure due to the abberant hormonal milieu [69,70], (2) metabolic alterations that regulate decidualization [71], (3) compromised PR functions led to total failure of the uterus in supporting embryo implantation [72], and (4) altered intrauterine microenvironment via deregulation of local inflammatory mediators [73,74]. In clinical practice, PCOS is related to a higher risk of miscarriage and adverse pregnancy outcomes [75], though its effect on IVF and FET cycles remains controversial. The advantages of GnRHa administration include its ability to ameliorate hyperandrogenism and inhibit the function of the GnRH-HCG axis while also reducing endometrial inflammation and enhancing the expression of endometrial adhesion molecules [76]. We found herein substantial improvements in CPR, LBR, and MR (Figure 4 and Supplementary Figure S6) among PCOS women pretreated with GnRHa prior to transfer, though the difference in implantation rate did not reach significance. Our findings support the hypothesis that pituitary suppression may effectively alleviate the detrimental effects on implantation in females diagnosed with this syndrome, particularly in cases where metabolic syndrome or hyperandrogenism is present [25].

However, it is important to note that the effects of GnRHa on the immune and endocrine systems remain unclear and may vary depending on the individual and their specific condition. In women with ovulatory disorders, pituitary down-regulation can facilitate the restoration of a normal endometrial cycle by reverting the endometrium to its original status. Additionally, it provides temporary relief from associated abnormalities like hyperandrogenism or excessive inflammation caused by overexposure to estrogen. However, these problems are usually absent in women who experience regular menstrual periods and do not have an ovulatory disorder. Therefore, from a pathophysiological perspective, GnRHa may not have a beneficial effect on this particular group of patients. In line with the above hypothesis, no significant differences in pregnancy outcomes were found among normo-ovulatory women with regular menstruation in our subset meta-analysis (Figure 4 and Supplementary Figure S7). In women with normal functioning ovaries, the use of GnRHa has not been able to demonstrate sufficient efficacy and should be considered with caution since pretreatment may result in a doubling of expenditures [11] without a commensurate improvement in pregnancy outcomes. The choice of GnRHa, as well as the dosage and duration of treatment, should be carefully assessed according to each patient’s characteristics and medical history.

Interestingly, in addition to GnRH agonists, the use of oral contraceptive pills (OCPs) and GnRH antagonists in artificial reproductive technology (ART) has been reported as being more patient-friendly, although their efficacy remains controversial [77,78]. Additional research is imperative to ascertain the impact of various down-regulation approaches on the success of infertility treatments in subpopulations of women with diverse underlying medical conditions.

## 5. Conclusions

Pituitary suppression with GnRHa during AC-FET cycles could demonstrate a beneficial role in certain patient settings, in which long-term suppression and the use of depot GnRHa protocols supposedly provide better pregnancy outcomes. Individuals with PCOS benefit from GnRHa pretreatment, while this FET protocol should be carefully considered in ovulatory women with regular menstruation. The discrepancies between RCTs and real-world data are the main limitation of this study and call for a more rigorous investigation.

## Figures and Tables

**Table 2:** Subgroup analyses of interested FET outcomes in women with and without GnRHa pretreatment. (A) Subgroup analysis of study design. (B) Subgroup analysis of the type of GnRHa (C) Subgroup analysis of the duration of pituitary suppression with GnRHa.

Outcomes		I2	OR (95% CI)	*p*	*p*-Value for Subgroup Differences ^κ^
(A) Subgrouping: Study design					
CPR	RCTs (k = 14)	18.00%	1.09 (0.87–1.36)	0.45	0.13
	Observational studies (k = 30)	77.20%	1.33 (1.13–1.55)	<0.001 *	
IR	RCTs (k = 12)	0.00%	1.01 (0.85–1.20)	0.88	0.04 *
	Observational studies (k = 15)	80.80%	1.40 (1.13–1.73)	0.004	
LBR	RCTs (k = 5)	37.80%	1.14 (0.68–1.41)	0.63	0.44
	Observational studies (k = 22)	81.30%	1.34 (1.06–1.70)	0.02	
MR	RCTs (k = 11)	44.60%	0.75 (0.31–1.82)	0.49	0.70
	Observational studies (k = 25)	56.70%	0.89 (0.71–1.10)	0.26	
(B) Subgrouping: Type of GnRHa protocol					
CPR	Depot GnRHa (k = 29)	72.10%	1.25 (1.08–1.44)	0.004 *	0.15
	Daily GnRHa (k = 14)	0.00%	1.11 (1.02–1.21)	0.02 *	
IR	Depot GnRHa (k = 17)	78.90%	1.28 (1.02–1.59)	0.03 *	0.51
	Daily GnRHa (k = 10)	1.70%	1.17 (0.96–1.42)	0.08	
LBR	Depot GnRHa (k = 19)	72.10%	1.19 (1.04–1.37)	0.02 *	0.75
	Daily GnRHa (k = 7)	26.80%	1.14 (0.86–1.52)	0.18	
MR	Depot GnRHa (k = 25)	48.20%	0.93 (0.75–1.15)	0.48	0.16
	Daily GnRHa (k = 10)	0.00%	1.10 (0.95–1.28)	0.16	
(C) Subgrouping: Duration of pituitary suppression with GnRHa					
CPR	Within one cycle (k = 37)	61.30%	1.17 (1.04–1.32)	0.01 *	0.003 *
	More than one cycle (k = 6)	66.40%	2.00 (1.29–3.10)	0.01 *	
IR	Within one cycle (k = 24)	56.70%	1.16 (1.02–1.32)	0.03 *	0.002 *
	More than one cycle (k = 3)	64.40%	2.07 (0.97–4.43)	0.05 *	
LBR	Within one cycle (k = 24)	77.80%	1.28 (1.03–1.60)	0.03 *	0.58
	More than one cycle (k = 2)	37.90%	1.42 (0.24–8.39)	0.24	
MR	Within one cycle (k = 32)	53.70%	0.87 (0.69–1.09)	0.21	0.54
	More than one cycle (k = 3)	72.60%	0.50 (0.01–24.11)	0.52	

^κ^: All subgroup analyses were conducted using a random-effects model, chosen in response to the significant heterogeneity observed within the overall study population. *: Statistically significant. CPR: Clinical Pregnancy Rate; GnRHa: Gonadotropin Releasing Hormone agonist; IR: Implantation Rate; LBR: Live Birth Rate; MR: Miscarriage Rate; RCT: Randomized Controlled Trial.

## Data Availability

Dataset available on request from the authors.

## Supplementary Materials

The following supporting information can be downloaded at: https://www.mdpi.com/article/10.3390/biomedicines12040760/s1, Table S1: PRISMA 2020 checklist [79]; Table S2: Detailed search strategy for electronic database searches (Searches performed on 30 August 2022). Table S3: Inclusion and exclusion criteria of the studies in the systematic review and meta-analysis; Table S4: Sensitive analysis for outcomes with publication biases; Figure S1: Baujat plot for sources of heterogeneity in overall infertile population (Clinical Pregnancy Rate); Figure S2: Baujat plot for sources of heterogeneity in overall infertile population (Miscarriage Rate); Figure S3: Baujat plot for sources of heterogeneity in overall infertile population (Live Birth Rate); Figure S4: Forest plots of meta-analysis for pregnancy outcomes following AC-FET cycles with and without GnRHa pretreatment: subgroups of daily and depot GnRHa protocols; Figure S5: Forest plots of meta-analysis for pregnancy outcomes following AC-FET cycles with and without GnRHa pretreatment: subgroups of GnRHa administration duration; Figure S6: Forest plots of meta-analysis for pregnancy outcomes following AC-FET cycles with and without GnRHa pretreatment in PCOS patients; Figure S7: Forest plots of meta-analysis for pregnancy outcomes following AC-FET cycles with and without GnRHa pretreatment in ovulatory women with regular cycles.
