# Supplementary material for: Pituitary Suppression with Gonadotropin-Releasing Hormone Agonist Prior to Artificial Endometrial Preparation in Frozen–Thawed Embryo Transfer Cycles: A Systematic Review and Meta-Analysis of Different Protocols and Infertile Populations
Source: Biomedicines. 2024 Mar 29;12(4):760. doi: 10.3390/biomedicines12040760 (PMC11048410; doi:10.3390/biomedicines12040760)
Supplement: Supplementary file 1 [file biomedicines-12-00760-s001.zip › biomedicines-2878236-supplementary.pdf]

Supplementary Table S1. PRISMA 2020 checklist

Section and Topic	Item #	Checklist item	Location where item is reported
**TITLE**			
Title	1	Identify the report as a systematic review.	Line 1-5
**ABSTRACT**			
Abstract	2	See PRISMA 2020 for Abstracts checklist.	Line 18-47
**INTRODUCTION**			
Rationale	3	Describe the rationale for the review in the context of existing knowledge.	Line 42-96
Objectives	4	Provide an explicit statement of the objective(s) or question(s) the review addresses.	Line 91-96
**METHODS**			
Eligibility criteria	5	Specify the inclusion and exclusion criteria for the review and how studies were grouped for the syntheses.	Line 99-131
Information sources	6	Specify all databases, registers, websites, organisations, reference lists and other sources searched or consulted to identify studies. Specify the date when each source was last searched or consulted.	Line 104-110
Search strategy	7	Present the full search strategies for all databases, registers and websites, including any filters and limits used.	Line 99-131 and Supplementary Table 2
Selection process	8	Specify the methods used to decide whether a study met the inclusion criteria of the review, including how many reviewers screened each record and each report retrieved, whether they worked independently, and if applicable, details of automation tools used in the process.	Line 111-123
Data collection process	9	Specify the methods used to collect data from reports, including how many reviewers collected data from each report, whether they worked independently, any processes for obtaining or confirming data from study investigators, and if applicable, details of automation tools used in the process.	Line 117-131
Data items	10a	List and define all outcomes for which data were sought. Specify whether all results that were compatible with each outcome domain in each study were sought (e.g., for all measures, time points, analyses), and if not, the methods used to decide which results to collect.	Line 127-131
	10b	List and define all other variables for which data were sought (e.g., participant and intervention characteristics, funding sources). Describe any assumptions made about any missing or unclear information.	Line 127-131
Study risk of bias assessment	11	Specify the methods used to assess risk of bias in the included studies, including details of the tool(s) used, how many reviewers assessed each study and whether they worked independently, and if applicable, details of automation tools used in the process.	Line 136-148 and Figure 2
Effect measures	12	Specify for each outcome the effect measure(s) (e.g., risk ratio, mean difference) used in the synthesis or presentation of results.	Line 135-162
Synthesis methods	13a	Describe the processes used to decide which studies were eligible for each synthesis (e.g., tabulating the study intervention characteristics and comparing against the planned groups for each synthesis (item #5)).	Line 135-162
	13b	Describe any methods required to prepare the data for presentation or synthesis, such as handling of missing summary statistics, or data conversions.	Line 135-162
	13c	Describe any methods used to tabulate or visually display results of individual studies and syntheses.	Line 135-162
	13d	Describe any methods used to synthesize results and provide a rationale for the choice(s). If meta-analysis was performed, describe the model(s), method(s) to identify the presence and extent of statistical heterogeneity, and software package(s) used.	Line 135-162
	13e	Describe any methods used to explore possible causes of heterogeneity among study results (e.g., subgroup analysis, meta-regression).	Line 135-162
	13f	Describe any sensitivity analyses conducted to assess robustness of the synthesized results.	Line 135-162
Reporting bias assessment	14	Describe any methods used to assess risk of bias due to missing results in a synthesis (arising from reporting biases).	Line 135-162
Certainty assessment	15	Describe any methods used to assess certainty (or confidence) in the body of evidence for an outcome.	Line 135-162
**RESULTS**			
Study selection	16a	Describe the results of the search and selection process, from the number of records identified in the search to the number of studies included in the review, ideally using a flow diagram.	Line 156-164, Figure 2, Supplementary Table 2
	16b	Cite studies that might appear to meet the inclusion criteria, but which were excluded,	Line 170-180

Section and Topic	Item #	Checklist item	Location where item is reported
		and explain why they were excluded.	
Study characteristics	17	Cite each included study and present its characteristics.	Table 1
Risk of bias in studies	18	Present assessments of risk of bias for each included study.	Line 197-202, and Figure 2
Results of individual studies	19	For all outcomes, present, for each study: (a) summary statistics for each group (where appropriate) and (b) an effect estimate and its precision (e.g., confidence/credible interval), ideally using structured tables or plots.	Line 203-278
Results of syntheses	20a	For each synthesis, briefly summarise the characteristics and risk of bias among contributing studies.	Line 203-278
	20b	Present results of all statistical syntheses conducted. If meta-analysis was done, present for each the summary estimate and its precision (e.g., confidence/credible interval) and measures of statistical heterogeneity. If comparing groups, describe the direction of the effect.	Line 203-278
	20c	Present results of all investigations of possible causes of heterogeneity among study results.	Line 203-278 and supplementary table 4
	20d	Present results of all sensitivity analyses conducted to assess the robustness of the synthesized results.	Line 203-278 and supplementary table 4, Supplementary Figure 1, 2 and 3
Reporting biases	21	Present assessments of risk of bias due to missing results (arising from reporting biases) for each synthesis assessed.	Line 203-278 and supplementary table 4, Supplementary Figure 1, 2 and 3
Certainty of evidence	22	Present assessments of certainty (or confidence) in the body of evidence for each outcome assessed.	Line 203-278
**DISCUSSION**			
Discussion	23a	Provide a general interpretation of the results in the context of other evidence.	Line 280-285
	23b	Discuss any limitations of the evidence included in the review.	Line 317-389
	23c	Discuss any limitations of the review processes used.	Line 340-373
	23d	Discuss implications of the results for practice, policy, and future research.	Line 360-373
**OTHER INFORMATION**			
Registration and protocol	24a	Provide registration information for the review, including register name and registration number, or state that the review was not registered.	Line 132-134
	24b	Indicate where the review protocol can be accessed, or state that a protocol was not prepared.	Line 132-134
	24c	Describe and explain any amendments to information provided at registration or in the protocol.	
Support	25	Describe sources of financial or non-financial support for the review, and the role of the funders or sponsors in the review.	Line 424
Competing interests	26	Declare any competing interests of review authors.	Line 427
Availability of data, code and other materials	27	Report which of the following are publicly available and where they can be found: template data collection forms; data extracted from included studies; data used for all analyses; analytic code; any other materials used in the review.	Supplementary Table 2

From: Page MJ, McKenzie JE, Bossuyt PM, Boutron I, Hoffmann TC, Mulrow CD, et al. The PRISMA 2020 statement: an updated guideline for reporting systematic reviews. BMJ 2021;372:n71. doi: 10.1136/bmj.n71

Supplementary Table S2. Detailed search strategy for electronic database searches
(Searches performed on 30th August 2022)

No.	Databases (4)	Search term	Total results (1333)
1	Google Scholar	(pituitary suppression OR GNRHa OR gonadotropin releasing hormone agonist) AND (FET OR frozen-thawed embryo transfer) AND (Artificial cycle OR HRT OR Hormonal replacement therapy OR HRC OR Hormonal replacement cycle)	1129
2	EMBASE	(pituitary suppression OR GNRHa OR gonadotropin releasing hormone agonist) AND (FET OR frozen-thawed embryo transfer) AND (Artificial cycle OR HRT OR Hormonal replacement therapy OR HRC OR Hormonal replacement cycle)	103
3	PubMed	(pituitary suppression OR GNRHa OR gonadotropin releasing hormone agonist) AND (FET OR frozen-thawed embryo transfer) AND (Artificial cycle OR HRT OR Hormonal replacement therapy OR HRC OR Hormonal replacement cycle)	65
4	Cochrane	(pituitary suppression OR GNRHa OR gonadotropin releasing hormone agonist) AND (FET OR frozen-thawed embryo transfer) AND (Artificial cycle OR HRT OR Hormonal replacement therapy OR HRC OR Hormonal replacement cycle) in Title Abstract Keyword	42

Supplementary Table S3: Inclusion and exclusion criteria of the studies in the systematic review and meta-analysis

ID	Author	Inclusion Criteria	Exclusion Criteria
1	Simon A. (1998)	(i) Women with **functioning ovaries** **(ii)** Autologous FET	NR
2	Prato L. D. (2002)	(i) Women from 25 to 38 years old (ii) Tubal, idiopathic, or male factors (iii) Autologous FET	(i) Women with < 2 embryos cryopreserved (ii) Women with all embryos cryopreserved because of ovarian hyperstimulation risk
3	El-Toukhy T. (2004)	(i) Women with regular menstrual cycle (ii) Autologous FET	(i) FET cycle with donated embryo(s)
4	Davar R. (2007)	(i) Women < 30 years old (ii) Regular menstruation (iii) Undergoing autologous FET	NR
5	Niu Z. (2013)	(i) Women aged 38 years or less (ii) History of ≤ 1 past failed FET (iii) Normal uterine cavity (iv) Having adenomyosis	(i) Presence of hydrosalpinxes (ii) Severe endometriosis (iii) History of myomectomy
6	Vijiver A. (2014)	(i) Women aged 39 years or less (ii) Underwent one or more FER cycle with same protocol for endometrial preparation (no cross-over)	(i) FET cycle with donated embryo(s)
7	Nekoo E. A. (2015)	(i) Infertile women from male factor (ii) 20-37 years old (iii) Regular menstruation (iv) Undergoing autologous FET	NR
8	Hebisha S. (2016)	(i) Women undergoing autologous FET with long agonist COS protocol	NR
9	Guo S. (2016)	(i) Age =<40 (ii) Undergoing FET (iii) Cleavages transferred	(i) Age>40 (ii) Concurrent endometriosis or fibroids (iii) Blastocyst transplantation
10	Tsai H. W. (2017)	(i) Infertile women with polycystic ovarian syndrome (PCOS) (ii) From 20 to 45 years old (iii) Autologous FET	(i) Using hormonal therapy (ii) Severe physical or mental illness (iii) Had pregnancy or breastfeeding in 6 weeks before (iv) Congenital adrenal hyperplasia, Cushing's syndrome or androgen-selecting neoplasm
11	Kang J. (2018)	(i) Regular menstrual cycle (24–35 days) (ii) 21–45 years of age (iii) Autologous FET	(i) PCOS (ii) FET with PGT embryo(s) (iii) Oocytes donation (iv) Endometriosis stage IV or history of uterine synechiae
12	Movahedi. S. (2018)	(i) Women from 25 to 38 years old (ii) Functioning ovaries and normal uterus cavity (iii) Autologous FET	(i) Age > 39 years old (ii) FSH > 11 (iii) Endometriosis or hypothalamic amenorrhea
13	Samsami A. (2018)	(i) Women from 20 to 39 years old (ii) Autologous FET (iii) Women from GnRHa group had embryo(s) from long GnRHa COS cycle(s)	(i) High grade endometriosis (ii) Uterine myoma or adhesion (iii) BMI < 18 or > 29 kg/m2
14	Wageh A. (2018)	(i) PCOS (ii) autologous FET	NR
15	Xie D. (2018)	(i) Autologous FET cycles	(i) blastocyst embryo(s) (ii) repeated thin endometrium (<7 mm) (iii) women with karyotype abnormalities
16	Madani T. (2019)	(i) Age 20-37 (ii) Normal menstruation cycle (iii) BMI<30 (iv) First FET cycle	(i) Embryo donation (ii) Uterine malformation (iii) Hyperprolactinemia (iv) Thyroid disorders (v) Ovulation disorders (vi) History of recurrent miscarriage (vii) Tuberculosis (viii) Severe endometriosis
17	Mehrafza M. (2019)	(i) All FET case	(i) Thin endometrium (ii) Homogenous hyperechogenic endometrium
18	Wang Z. (2019)	(i) Patients with endometrial polyps and undergoing FET	NR
19	Aghahoseini M. (2020)	(i) Infertile women 18-40 years old (ii) BMI ≤ 30 kg/m^2 (iii) PCOS diagnosed using Rotterdam criteria	(i) Being treated with hormones or drugs (ii) Having severe physical or mental illness (iii) Having congenital adrenal hyperplasia or androgen-secreting neoplasm (iv) Having uterine abnormalities
20	An J. (2020)	(i) 21-40 years old (ii) Regular menstruation (iii) ≥ 2 cryopreserved embryos	(i) FET cycles with PGT (ii) Endometriosis, uterine malformations, hydrosalpinx or a history of intrauterine adhesions
21	Davar R. (2020)	(i) History of idiopathic RIF (at least two implantation failures)	(i) Endometrial polyp, uterine myoma, and uterine anomaly
22	Dong M. (2020)	(i) 38-45 years old	(i) FET with PGT (ii) Blastocysts from cryopreserved or donated oocytes

		(ii) First autologous FET cycles with ICSI embryo(s)	(iii) Thin endometrium on FET day<br>(iv) Non-AC cycles<br>(v) Fresh cycle transplantation, history of repeated abortions, congenital uterine malformations
23	Guerrero-Vargas J. J. (2020)	(i) Autologous FET cycles	(i) Progesterone >1.5 ng/dl on day of triggering in natural cycle<br>(ii) Untreated endometrial fluid, polyps or submucosal fibroids<br>(iii) No inflammation or infection of the genital tract
24	Naserpoor L. (2020)	(i) Women undergoing FET	NR
25	Qi Q. (2020)	(i) All women undergoing FET using AC protocol	NR
26	Li M. (2021)	(i) Women ≤ 45 years old undergoing 1st autologous FET cycle<br>(ii) Having adenomyosis	(i) Malformations of reproductive system without therapy<br>(ii) Hydrosalpinx, PCOS, endometriosis, malignant diseases of reproductive system<br>(iii) Parental chromosomal abnormality
27	Liu X. (2021)	(i) Women undergoing 1st autologous FET cycle<br>(ii) Having PCOS diagnosed using Rotterdam criteria	(i) Women with multiple FET
28	Luo L. (2021)	(i) Women 20-40 years old<br>(ii) PCOS diagnosed using Rotterdam criteria<br>(iii) < 3 blastocysts transferred	(i) Women with other causes of ovulation dysfunction and hyperandrogenism<br>(ii) Anatomical uterine abnormalities<br>(iii) Women with PGT embryo(s) or contraindications to AC therapy<br>(iv) Endometriosis, adenomyosis, RIF, recurrent pregnancy loss (three or more times)<br>(v) Parental chromosomal abnormalities<br>(vi) Thin endometrium after ≥ days of estrogen administration
29	Salama K. M.(2021)	(i) Age 22-40 years<br>(ii) BMI < 36 kg/m2	(i) Gross uterine and tubal pathology<br>(ii) Endometrial thickness ≤ 7 mm at the time of embryo transfer<br>(iii) Poor quality of embryos after thawing<br>(iv) Refusal to participate in the study at any step of the cycle.
30	Salemi S. (2021)	(i) Age <37<br>(ii) PCOS<br>(iii) Autologous FET<br>(iv) Normal uterine cavity	(i) Testicular sperm ICSI<br>(ii) Basal FSH > 12 IU/L<br>(iii) Egg donor or surrogates<br>(iv) Hydrosalpinx, uterine anomalies, submucousal myoma, blastocyst transfer
31	Siristatidis C. (2021)	(i) Age 25–42 years<br>(ii) BMI ≤ 35 and ≥19<br>(iii) Normo-ovulatory patients	(i) Poor ovarian response according to the Bologna criteria<br>(ii) PCOS patients according to the Rotterdam criteria<br>(iii) Patients using donor oocytes and gestational carriers
32	Xu J. (2021)	(i) Women aged 20–38 years<br>(ii) All embryos were vitrified with at least two high-quality day 3 embryos<br>(iii) Undergoing ET for the first time.	(i) FET cycles after pre-implantation genetic testing<br>(ii) Patients with congenital or acquired uterine malformations, intrauterine adhesion, laparoscopic findings suggesting endometriosis, ultrasound findings suggesting adenomyosis, intramural uterine leiomyoma (≥3 cm), submucosal fibroids, scarred uterus, endometrial polyp, hydrosalpinx, PCOS, recurrent abortions (defined as three or more previous spontaneous pregnancy losses)<br>(iii) Abnormal results on parental karyotyping<br>(iv) Medical conditions that contraindicated assisted reproductive technology (ART) treatment or pregnancy
33	Zheng Q. Z. (2021)	(i) Completed FET cycles	(i) No embryos for transferred<br>(ii) FET with PGT-A<br>(iii) Cycles with mixed embryos from different ovarian stimulation cycles or different embryo stage<br>(iv) Cycles lost to follow up
34	Eleftheriadou A. (2022)	(i) First FET cycles	(i) FET with PGT
35a	Xia L. (2022)*	(i) Completed AC-FET cycles without a history of implantation failure(s)	(i) Endometriosis<br>(ii) Adenomyosis<br>(iii) PCOS<br>(iv) Endometritis<br>(v) Intrauterine adhesions<br>(vi) Uterine malformation<br>(vii) Untreated hydrosalpinx
35b	Xia L. (2022)**	(i) Completed AC-FET cycles with 1 previous failed implantation	(i) Endometriosis<br>(ii) Adenomyosis<br>(iii) PCOS<br>(iv) Endometritis<br>(v) Intrauterine adhesions<br>(vi) Uterine malformation<br>(vii) Untreated hydrosalpinx
35c	Xia L. (2022)***	(i) Completed AC-FET cycles with > 1 previous failed implantation	(i) Endometriosis<br>(ii) Adenomyosis<br>(iii) PCOS<br>(iv) Endometritis<br>(v) Intrauterine adhesions<br>(vi) Uterine malformation

			(vii) Untreated hydrosalpinx
36	Li L. (2022)	(i) 18-40 years old<br>(ii) Regular menstrual cycle of 26-35 days<br>(iii) Undergoing autologous AC-FET cycles	(i) Contraindication to estrogen or progesterone (prior thrombosis, hormone-sensitive malignancy, porphyria...)<br>(ii) Intrauterine adhesions<br>(iii) Congenital uterine abnormalities<br>(iv) Recipients of oocyte donation programs
37	Pan D. (2022)	(i) Patients 36-43 years old<br>(ii) Undergoing third ET attempt or higher	(i) Adenomyosis or endometriosis<br>(ii) Thin endometrium < 7mm on day of ET<br>(iii) Recipients of oocyte donation program
38	Wang Y. (2022)	(i) PCOS patients undergoing AC-FET	(i) Other causes of hyperandrogenism and ovulation dysfunction<br>(ii) Congenital or acquired uterine malformations<br>(iii) Endometriosis and adenomyosis<br>(iv) Intrauterine adhesions<br>(v) History of recurrent miscarriage<br>(vi) FET cycles with PGT
39	Gan R. X. (2022)	(i) Infertile women with cesarean scar	(i) > 40 years of age at oocyte retrieval<br>(ii) History of multiple cesarean scars and impaired cesarean scar healing<br>(iii) Recurrent spontaneous abortion, recurrent implantation failure<br>(iv) Reimplantation genetic testing<br>(v) Previous uterine myomectomy or operative hysteroscopy for intrauterine adhesions, thin endometrium (< 7 mm on the day of embryo transfer), untreated hydrosalpinx, adenomyosis, autoimmune or endocrine disease<br>(vi) Missing records in the electronic database
40	Mo M. (2022)	(i) Women with a clear history of IUA and underwent an intrauterine adhesion serration procedure	(i) Cycle with no autologous embryo(s) or cycle with PGT embryo<br>(ii) Cycle with embryo coming from different IVF cycles<br>(iii) Cycle lost to follow up
41	Liu Y. (2022)	(i) Patients with a 2-time <8mm endometrium on the day of oocyte retrieval or day of P4 administration	(i) Endometrium-related diseases including uterine malformations, uterine myoma, endometrial polyps, intrauterine adhesion, genital tuberculosis, and hydrosalpinx
AC: artificial cycle, HRT: hormonal replacement therapy, NR: Non-Reported			

Supplementary Table S4. Sensitive analysis for outcomes with publication biases.

(A) In general population

Outcome	Overall									Exclusion of outliers								
	Fixed effect model			Random effect model			I2	p	Egger's test p-value	Fixed effect model			Random effect model			I2	p	Egger's test p-value
	OR/MD	95%CI	p	OR/MD	95%CI	p				OR/MD	95%CI	p	OR/MD	95%CI	p			
**CPR**	1.1338	[1.0825; 1.1875]	<0.001	1.2664	[1.1164; 1.4364]	<0.001	69.4% [58.3%; 77.5%]	<0.001	0.013	1.1700	[1.1106; 1.2326]	<0.001	1.1942	[1.0959; 1.3013]	<0.001	38.5% [10.3%; 57.9%]	0.0073	0.2473
**IR**	1.187	[1.1288; 1.2482]	<0.001	1.2446	[1.0712; 1.4461]	0.006	70.3% [56.1%; 79.9%]	<0.001	0.3517	-	-	-	-	-	-	-	-	-
**MR**	1.0148	[0.9334; 1.1033]	0.7304	0.8581	[0.6839; 1.0768]	0.1799	52.9% [31.2%; 67.8%]	<0.001	0.0065	0.9189	[0.8248; 1.0238]	0.1252	0.9022	[0.7809; 1.0423]	0.1561	19.2% [0.0%; 48.0%]	0.1702	0.0920
**LBR**	1.1003	[1.0474; 1.1559]	<0.001	1.3088	[1.0717; 1.5985]	0.01	78.0% [68.4%; 84.6%]	<0.001	0.002	1.1865	[1.1143; 1.2633]	<0.001	1.2230	[1.0866; 1.3767]	0.0025	52.3% [23.9%; 70.0%]	0.0016	0.1402

(B) In women with PCOS diagnosis

Outcome	Overall									Exclusion of outliers								
	Fixed effect model			Random effect model			I2	p	Egger's test p-value	Fixed effect model			Random effect model			I2	p	Egger's test p-value
	OR/MD	95%CI	p	OR/MD	95%CI	p				OR/MD	95%CI	p	OR/MD	95%CI	p			
**CPR**	1.2379	[1.0646; 1.4395]	0.0056	1.2346	[1.0062; 1.5148]	0.0446	29.1% [0.0%; 65.0%]	0.1687	0.4367	-	-	-	-	-	-	-	-	-
**IR**	1.2840	[1.0009; 1.6472]	0.0492	1.3502	[0.4631; 3.9370]	0.3508	71.4% [3.1%; 91.6%]	0.0302	0.2627	-	-	-	-	-	-	-	-	-
**MR**	0.7451	[0.5862; 0.9471]	0.0162	0.6577	[0.3277; 1.3202]	0.1981	44.9% [0.0%; 75.6%]	0.0795	0.1893	-	-	-	-	-	-	-	-	-
**LBR**	1.2199	[1.0476; 1.4206]	0.0105	1.2197	[0.9222; 1.6133]	0.1274	48.6% [0.0%; 79.6%]	0.0833	0.6499	-	-	-	-	-	-	-	-	-

(C) In women with reported regular menstruation

Outcome	Overall									Exclusion of outliers								
	Fixed effect model			Random effect model			I2	p	Egger's test p-value	Fixed effect model			Random effect model			I2	p	Egger's test p-value
	OR/MD	95%CI	p	OR/MD	95%CI	p				OR/MD	95%CI	p	OR/MD	95%CI	p			
**CPR**	1.5592	[1.3355; 1.8202]	<0.001	1.6285	[0.9784; 2.7106]	0.06	76.7% [53.5%; 88.3%]	<0.001	0.7394	-	-	-	-	-	-	-	-	-
**IR**	1.4225	[1.2169; 1.6627]	<0.001	1.3051	[0.8387; 2.0308]	0.1823	60.7% [3.7%; 83.9%]	0.0262	0.4821	-	-	-	-	-	-	-	-	-
**MR**	0.7523	[0.5512; 1.0269]	0.0730	0.5635	[0.1950; 1.6284]	0.2341	73.3% [42.6%; 87.6%]	0.0010	0.2307	-	-	-	-	-	-	-	-	-
**LBR**	1.7059	[1.4315; 2.0329]	<0.001	2.1262	[0.8213; 5.5045]	0.0970	86.6% [73.0%; 93.3%]	<0.001	0.2330	-	-	-	-	-	-	-	-	-

(A)

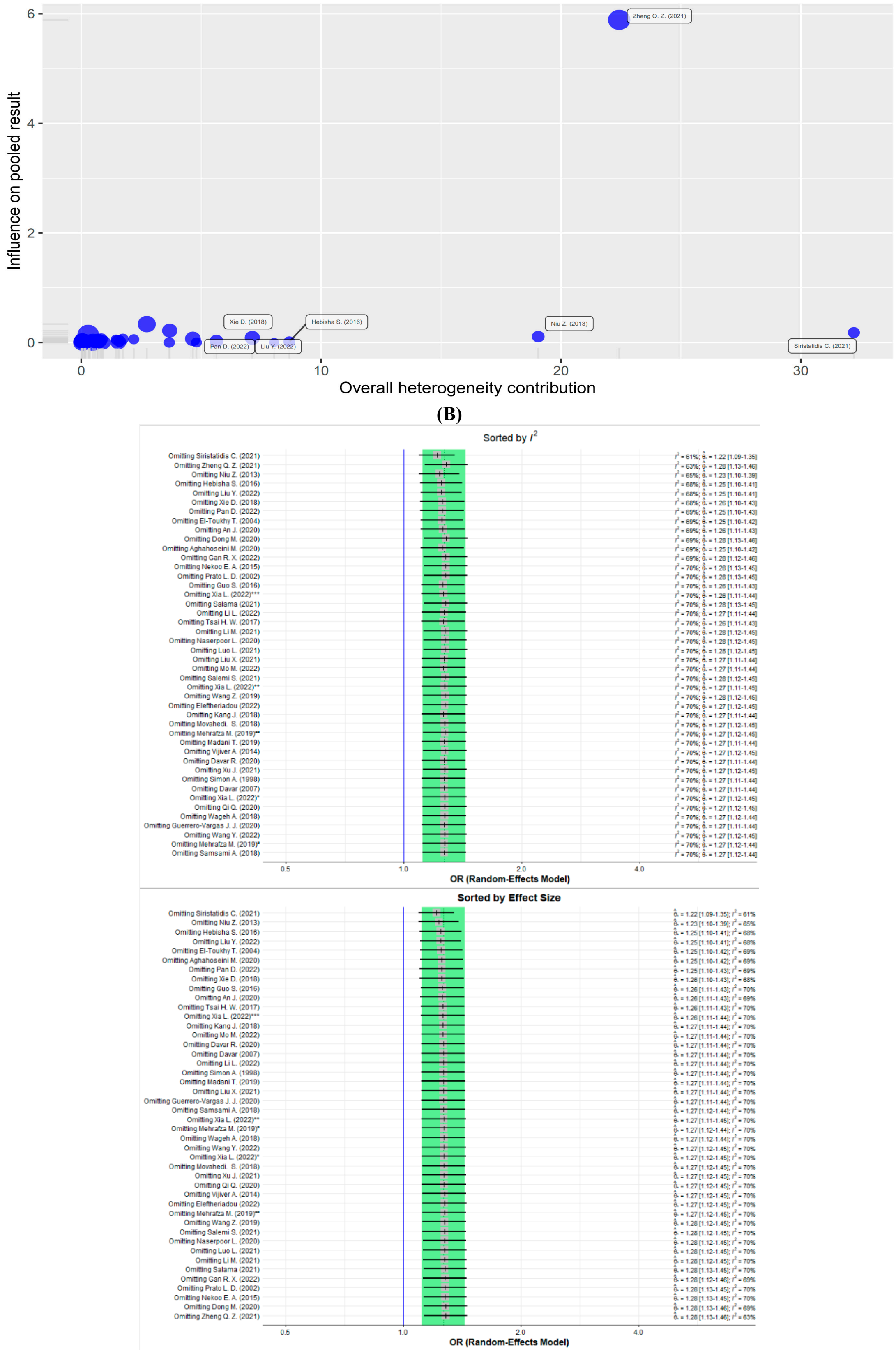

Supplementary Figure S1: Baujat plot for sources of heterogeneity in overall infertile population **(Clinical Pregnancy Rate). (A)** Baujat plot **(B)** Leave-One-Out Meta-Analysis Results sorted by I^2 and Effect Size

(A)

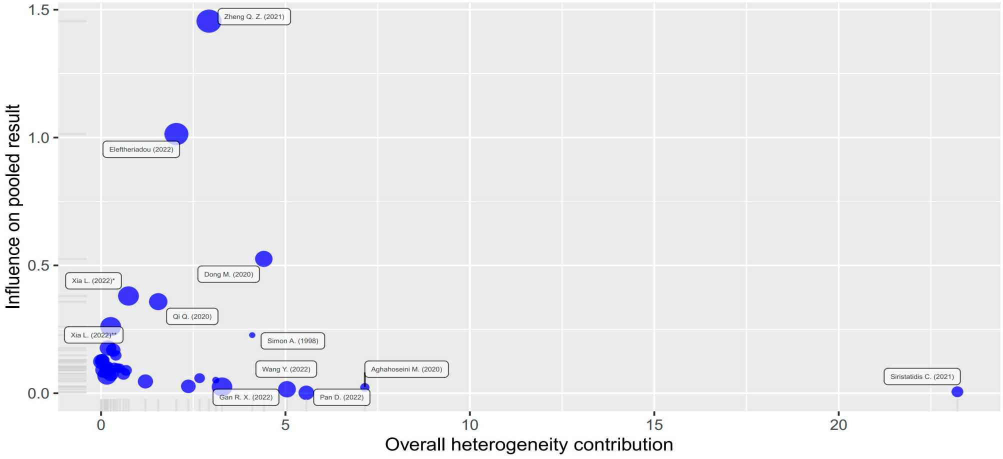

(B)

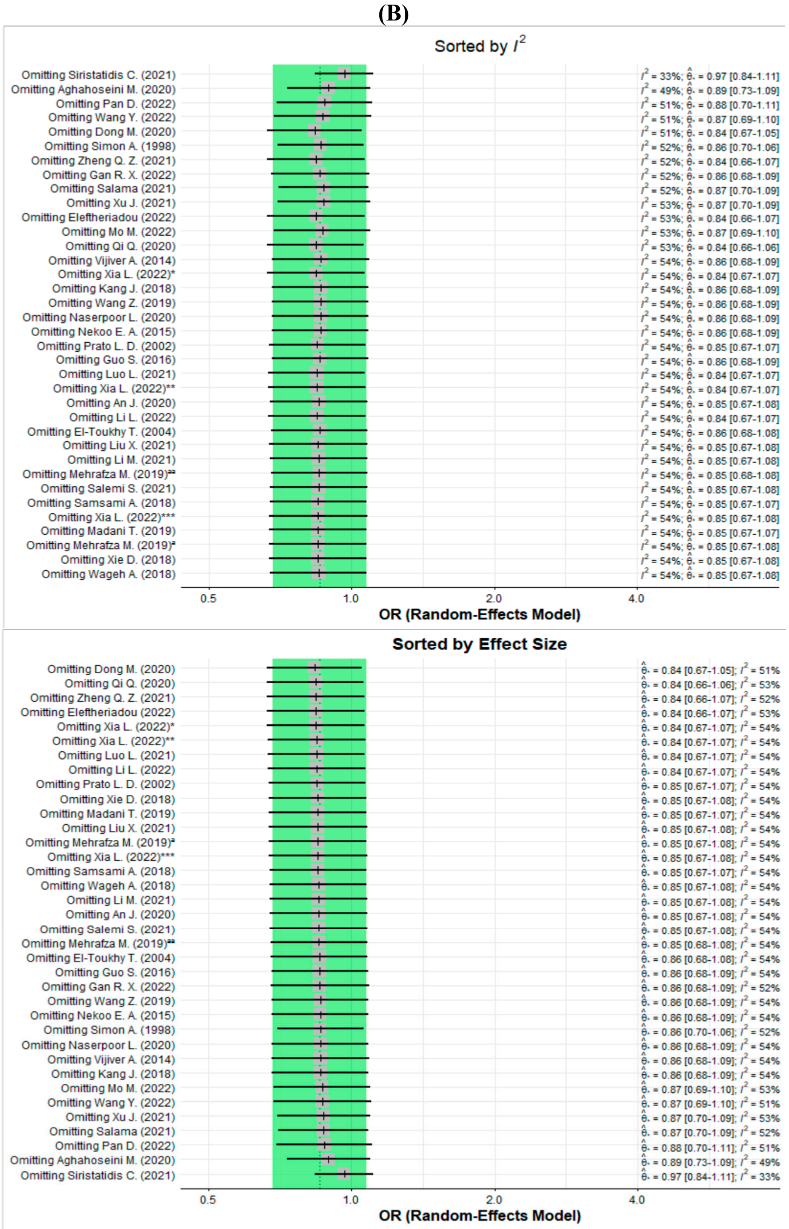

Supplementary Figure S2: Baujat plot for sources of heterogeneity in overall infertile population **(Miscarriage Rate).** **(A)** Baujat plot **(B)** Leave-One-Out Meta-Analysis Results sorted by I^2 and Effect Size

(A)

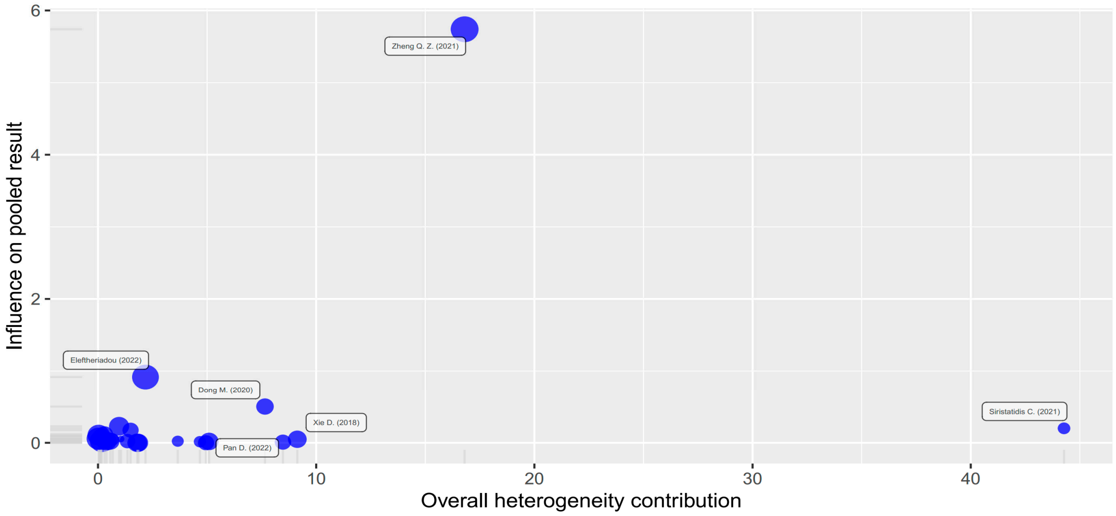

(B)

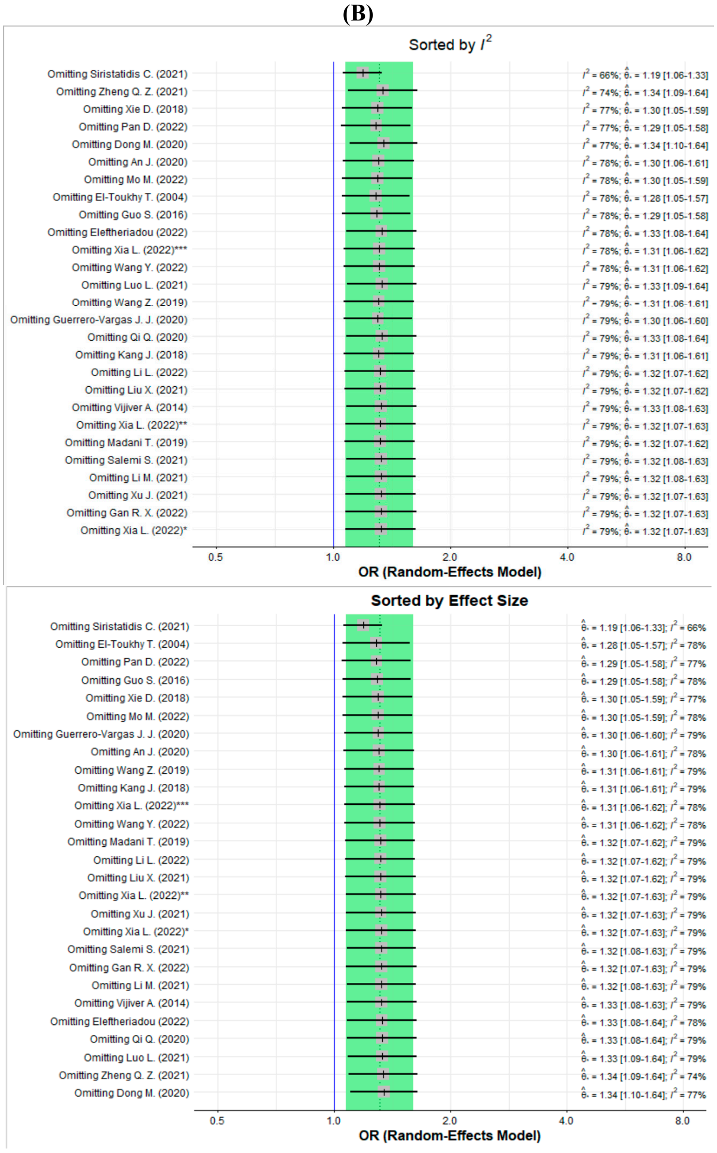

Supplementary Figure S3: Baujat plot for sources of heterogeneity in overall infertile population **(Live Birth Rate).** **(A)** Baujat plot **(B)** Leave-One-Out Meta-Analysis Results sorted by I^2 and Effect Size

(A) Clinical pregnancy rate

Study or Subgroup	Experimental Events	Experimental Total	Control Events	Control Total	Weight (common)	Weight (random)	Odds Ratio MH, Fixed + Random, 95% CI
Subgroup = Depot							
Simon A. (1998)	14	53	11	52	0.2%	1.0%	1.34 [0.54; 3.30]
Prato L. D. (2002)	28	142	34	141	0.8%	1.9%	0.77 [0.44; 1.36]
Niu Z. (2013)	100	194	36	145	0.6%	2.4%	3.22 [2.01; 5.16]
Nekoo E. A. (2015)	20	93	24	83	0.6%	1.5%	0.67 [0.34; 1.34]
Hebisha S. (2016)	72	110	42	100	0.5%	2.0%	2.62 [1.50; 4.57]
Guo S. (2016)	39	76	16	44	0.3%	1.3%	1.84 [0.86; 3.95]
Tsai H. W. (2017)	21	29	18	31	0.1%	0.8%	1.90 [0.64; 5.60]
Xie D. (2018)	126	252	281	751	2.1%	3.5%	1.67 [1.25; 2.23]
Mehrafza M. (2019)[aa]	27	93	31	103	0.6%	1.8%	0.95 [0.51; 1.76]
Wang Z. (2019)	47	92	204	396	1.1%	2.5%	0.98 [0.62; 1.55]
Aghahoseini M. (2020)	38	88	24	90	0.4%	1.7%	2.09 [1.11; 3.92]
An J. (2020)	401	975	107	338	2.8%	3.6%	1.51 [1.16; 1.96]
Dong M. (2020)	90	268	370	996	3.1%	3.5%	0.86 [0.64; 1.14]
Qi Q. (2020)	147	303	1353	2936	3.9%	3.8%	1.10 [0.87; 1.40]
Li M. (2021)	65	160	77	181	1.3%	2.6%	0.92 [0.60; 1.42]
Liu X. (2021)	387	514	363	514	2.7%	3.5%	1.27 [0.96; 1.67]
Luo L. (2021)	109	172	111	171	1.2%	2.5%	0.94 [0.60; 1.45]
Salama (2021)	32	70	37	70	0.6%	1.6%	0.75 [0.39; 1.46]
Xu J. (2021)	39	65	41	68	0.5%	1.5%	0.99 [0.49; 1.98]
Zheng Q. Z. (2021)	768	1518	6186	11456	21.3%	4.5%	0.87 [0.78; 0.97]
Xia L. (2022)*	651	1165	610	1165	8.0%	4.2%	1.15 [0.98; 1.36]
Xia L. (2022)**	687	1133	637	1133	7.5%	4.2%	1.20 [1.01; 1.42]
Xia L. (2022)***	439	785	147	302	2.8%	3.6%	1.34 [1.03; 1.75]
Li L. (2022)	357	813	104	276	2.6%	3.5%	1.29 [0.98; 1.71]
Pan D. (2022)	142	290	68	194	1.2%	2.9%	1.78 [1.22; 2.58]
Wang Y. (2022)	223	309	837	1207	2.8%	3.5%	1.15 [0.87; 1.51]
Gan R. X. (2022)	399	846	483	1007	6.9%	4.1%	0.97 [0.81; 1.16]
Mo M. (2022)	72	155	116	294	1.3%	2.8%	1.33 [0.90; 1.97]
Liu Y. (2022)	23	43	12	54	0.1%	1.1%	4.03 [1.67; 9.68]
Total (fixed effect, 95% CI)		10806		24298	78.1%	--	1.12 [1.06; 1.18]
Total (random effects, 95% CI)					--	77.4%	1.25 [1.08; 1.44]
Heterogeneity: $Tau^2 = 0.0954$; $Chi^2 = 100.26$, df = 28 ($P < 0.01$); $I^2 = 72\%$							
Subgroup = Daily							
El-Toukhy T. (2004)	28	117	13	117	0.3%	1.4%	2.52 [1.23; 5.15]
Davar (2007)	3	30	2	30	0.1%	0.3%	1.56 [0.24; 10.05]
Vijiver A. (2014)	47	280	137	849	1.7%	3.0%	1.05 [0.73; 1.51]
Kang J. (2018)	55	113	20	49	0.4%	1.5%	1.38 [0.70; 2.71]
Movahedi. S. (2018)	9	60	7	40	0.2%	0.8%	0.83 [0.28; 2.45]
Samsami A. (2018)	18	109	16	107	0.4%	1.4%	1.12 [0.54; 2.34]
Wageh A. (2018)	19	37	29	58	0.3%	1.2%	1.06 [0.46; 2.41]
Madani T. (2019)	44	121	35	113	0.7%	2.0%	1.27 [0.74; 2.19]
Mehrafza M. (2019)[a]	33	100	31	103	0.6%	1.8%	1.14 [0.63; 2.07]
Davar R. (2020)	8	31	6	31	0.1%	0.6%	1.45 [0.44; 4.81]
Guerrero-Vargas J. J. (2020)	17	64	8	35	0.2%	0.9%	1.22 [0.47; 3.20]
Naserpoor L. (2020)	19	74	22	74	0.5%	1.4%	0.82 [0.40; 1.68]
Salemi S. (2021)	29	93	32	95	0.6%	1.8%	0.89 [0.48; 1.64]
Eleftheriadou (2022)	865	1949	1121	2658	15.7%	4.5%	1.09 [0.97; 1.23]
Total (fixed effect, 95% CI)		3178		4359	21.9%	--	1.11 [1.01; 1.23]
Total (random effects, 95% CI)					--	22.6%	1.11 [1.02; 1.21]
Heterogeneity: $Tau^2 = 0$; $Chi^2 = 7.64$, df = 13 ($P = 0.87$); $I^2 = 0\%$							
**Total (fixed effect, 95% CI)**		**13984**		**28657**	**100.0%**	**--**	**1.12 [1.07; 1.17]**
**Total (random effects, 95% CI)**					**--**	**100.0%**	**1.22 [1.09; 1.35]**

Heterogeneity: $Tau^2 = 0.0573$; $Chi^2 = 107.91$, df = 42 ($P < 0.01$); $I^2 = 61\%$
Test for subgroup differences (fixed effect): $Chi^2 = 0.02$, df = 1 ($P = 0.89$)
Test for subgroup differences (random effects): $Chi^2 = 2.05$, df = 1 ($P = 0.15$)

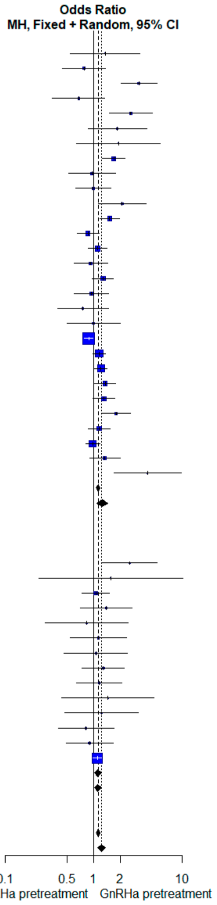

Supplementary Figure S4: Forest plots of meta-analysis for pregnancy outcomes following AC-FET cycles with and without GnRHa pretreatment: subgroups of daily and depot GnRHa protocols. **(A)** Clinical Pregnancy Rate. **(B)** Implantation Rate. **(C)** Live Birth Rate **(D)** Miscarriage Rate. *, **, and ***: three populations of infertile women were reported in the same study with matched or non-matched designs. a and aa: two different protocols were applied in the same study.

(B) Implantation rate

Study or Subgroup	Experimental Events	Experimental Total	Control Events	Control Total	Weight (common)	Weight (random)	Odds Ratio MH, Fixed + Random, 95% CI
**Subgroup = Depot**							
Simon A. (1998)	17	179	15	167	0.5%	2.5%	1.06 [0.51; 2.20]
Prato L. D. (2002)	31	299	36	301	1.2%	3.5%	0.85 [0.51; 1.42]
Niu Z. (2013)	127	390	45	280	1.3%	4.1%	2.52 [1.72; 3.70]
Nekoo E. A. (2015)	23	93	30	83	0.9%	2.8%	0.58 [0.30; 1.11]
Hebisha S. (2016)	142	322	60	285	1.3%	4.3%	2.96 [2.06; 4.24]
Guo S. (2016)	49	145	18	72	0.6%	2.9%	1.53 [0.81; 2.89]
Xie D. (2018)	155	575	333	1655	4.5%	5.0%	1.47 [1.18; 1.83]
Mehrafza M. (2019)[aa]	35	235	37	258	1.1%	3.5%	1.05 [0.63; 1.72]
An J. (2020)	585	2760	139	950	5.8%	5.1%	1.57 [1.28; 1.92]
Luo L. (2021)	145	245	127	214	2.0%	4.2%	0.99 [0.68; 1.44]
Salama (2021)	54	117	65	127	1.2%	3.5%	0.82 [0.49; 1.35]
Xu J. (2021)	60	126	58	128	1.1%	3.5%	1.10 [0.67; 1.80]
Xia L. (2022)*	830	1994	743	1968	15.6%	5.4%	1.18 [1.03; 1.34]
Xia L. (2022)**	879	1879	830	1889	15.8%	5.4%	1.12 [0.99; 1.28]
Xia L. (2022)***	560	1309	190	491	5.7%	5.0%	1.18 [0.96; 1.46]
Gan R. X. (2022)	461	1213	549	1400	11.3%	5.3%	0.95 [0.81; 1.11]
Liu Y. (2022)	29	86	15	107	0.3%	2.6%	3.12 [1.54; 6.32]
Total (fixed effect, 95% CI)		11967		10375	70.0%	--	1.22 [1.15; 1.29]
Total (random effects, 95% CI)					--	68.4%	1.28 [1.02; 1.59]
Heterogeneity: Tau^2 = 0.1532; Chi^2 = 75.89, df = 16 (P < 0.01); I^2 = 79%							
**Subgroup = Daily**							
El-Toukhy T. (2004)	43	269	26	258	0.8%	3.4%	1.70 [1.01; 2.86]
Davar (2007)	4	64	3	65	0.1%	0.8%	1.38 [0.30; 6.42]
Vijiver A. (2014)	47	239	137	733	1.9%	4.2%	1.06 [0.74; 1.54]
Kang J. (2018)	76	181	24	88	0.7%	3.2%	1.93 [1.11; 3.36]
Movahedi. S. (2018)	9	136	10	98	0.4%	1.8%	0.62 [0.24; 1.60]
Madani T. (2019)	58	302	54	280	1.6%	4.0%	0.99 [0.66; 1.50]
Mehrafza M. (2019)[a]	34	245	37	258	1.1%	3.5%	0.96 [0.58; 1.59]
Davar R. (2020)	8	52	6	57	0.2%	1.4%	1.55 [0.50; 4.80]
Salemi S. (2021)	54	198	48	201	1.2%	3.8%	1.20 [0.76; 1.88]
Eleftheriadou (2022)	1040	2261	1322	3004	22.0%	5.4%	1.08 [0.97; 1.21]
Total (fixed effect, 95% CI)		3947		5042	30.0%	--	1.11 [1.01; 1.22]
Total (random effects, 95% CI)					--	31.6%	1.17 [0.96; 1.42]
Heterogeneity: Tau^2 = 0.0454; Chi^2 = 9.15, df = 9 (P = 0.42); I^2 = 2%							
**Total (fixed effect, 95% CI)**		**15914**		**15417**	**100.0%**	**--**	**1.19 [1.13; 1.25]**
**Total (random effects, 95% CI)**					**--**	**100.0%**	**1.24 [1.07; 1.45]**

Heterogeneity: Tau^2 = 0.1103; Chi^2 = 87.57, df = 26 (P < 0.01); I^2 = 70%
Test for subgroup differences (fixed effect): Chi^2 = 2.74, df = 1 (P = 0.10)
Test for subgroup differences (random effects): Chi^2 = 0.43, df = 1 (P = 0.51)

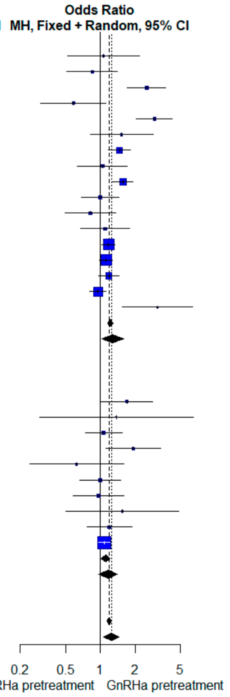

(C) Live birth rate

Study or Subgroup	Experimental Events	Experimental Total	Control Events	Control Total	Weight (common)	Weight (random)	Odds Ratio MH, Fixed + Random, 95% CI
**Subgroup = Depot**							
Guo S. (2016)	34	76	11	44	0.3%	1.4%	2.43 [1.07; 5.51]
Xie D. (2018)	105	252	220	751	2.1%	4.6%	1.72 [1.28; 2.32]
Wang Z. (2019)	37	92	126	396	0.9%	3.1%	1.44 [0.90; 2.30]
An J. (2020)	299	975	76	338	2.6%	4.7%	1.52 [1.14; 2.04]
Dong M. (2020)	47	268	240	996	2.8%	4.1%	0.67 [0.47; 0.95]
Qi Q. (2020)	106	303	1051	2936	4.3%	5.1%	0.97 [0.75; 1.24]
Li M. (2021)	38	160	43	181	1.0%	2.9%	1.00 [0.61; 1.65]
Liu X. (2021)	309	514	288	514	3.8%	5.1%	1.18 [0.92; 1.52]
Luo L. (2021)	85	172	92	171	1.6%	3.4%	0.84 [0.55; 1.28]
Xu J. (2021)	31	65	32	68	0.5%	1.9%	1.03 [0.52; 2.03]
Zheng Q. Z. (2021)	578	1518	4750	11456	22.9%	6.4%	0.87 [0.78; 0.97]
Xia L. (2022)*	527	1165	498	1165	9.1%	6.0%	1.11 [0.94; 1.30]
Xia L. (2022)**	570	1133	535	1133	8.9%	6.0%	1.13 [0.96; 1.33]
Xia L. (2022)***	357	785	117	302	3.1%	4.9%	1.32 [1.01; 1.73]
Li L. (2022)	246	813	72	276	2.5%	4.5%	1.23 [0.90; 1.67]
Pan D. (2022)	106	290	43	194	1.1%	3.5%	2.02 [1.34; 3.06]
Wang Y. (2022)	195	309	685	1207	3.4%	5.0%	1.30 [1.01; 1.69]
Gan R. X. (2022)	302	846	343	1007	6.7%	5.7%	1.07 [0.89; 1.30]
Mo M. (2022)	57	155	73	294	1.1%	3.4%	1.76 [1.16; 2.68]
Total (fixed effect, 95% CI)		9891		23429	78.7%	--	1.09 [1.03; 1.15]
Total (random effects, 95% CI)					--	81.6%	1.19 [1.04; 1.37]
Heterogeneity: Tau^2 = 0.0537; Chi^2 = 64.56, df = 18 (P < 0.01); I^2 = 72%							
**Subgroup = Daily**							
El-Toukhy T. (2004)	23	117	10	117	0.3%	1.5%	2.62 [1.19; 5.78]
Vijiver A. (2014)	41	275	127	839	1.8%	3.8%	0.98 [0.67; 1.44]
Kang J. (2018)	47	113	16	49	0.4%	1.8%	1.47 [0.73; 2.97]
Madani T. (2019)	38	121	31	113	0.7%	2.5%	1.21 [0.69; 2.13]
Guerrero-Vargas J. J. (2020)	11	64	3	35	0.1%	0.6%	2.21 [0.57; 8.54]
Salemi S. (2021)	20	93	21	95	0.5%	1.9%	0.97 [0.48; 1.93]
Eleftheriadou (2022)	716	1949	978	2658	17.4%	6.4%	1.00 [0.88; 1.13]
Total (fixed effect, 95% CI)		2732		3906	21.3%	--	1.04 [0.93; 1.16]
Total (random effects, 95% CI)					--	18.4%	1.14 [0.86; 1.52]
Heterogeneity: Tau^2 = 0.0281; Chi^2 = 8.2, df = 6 (P = 0.22); I^2 = 27%							
**Total (fixed effect, 95% CI)**		**12623**		**27335**	**100.0%**	**--**	**1.08 [1.03; 1.14]**
**Total (random effects, 95% CI)**					**--**	**100.0%**	**1.19 [1.06; 1.33]**

Heterogeneity: Tau^2 = 0.0460; Chi^2 = 73.48, df = 25 (P < 0.01); I^2 = 66%
Test for subgroup differences (fixed effect): Chi^2 = 0.64, df = 1 (P = 0.42)
Test for subgroup differences (random effects): Chi^2 = 0.10, df = 1 (P = 0.75)

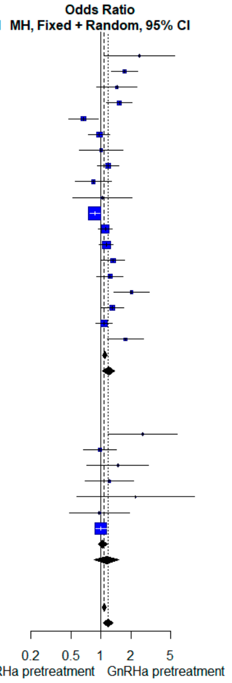

Supplementary Figure S4: (cont.)

(D) Miscarriage rate

Study or Subgroup	Experimental Events	Experimental Total	Control Events	Control Total	Weight (common)	Weight (random)	Odds Ratio MH, Fixed + Random, 95% CI
**Subgroup = Depot**							
Simon A. (1998)	7	14	0	11	0.0%	0.2%	23.00 [1.14; 465.16]
Prato L. D. (2002)	5	28	4	34	0.3%	0.8%	1.63 [0.39; 6.76]
Nekoo E. A. (2015)	2	20	4	24	0.3%	0.5%	0.56 [0.09; 3.40]
Guo S. (2016)	5	39	3	16	0.3%	0.7%	0.64 [0.13; 3.06]
Xie D. (2018)	18	126	39	281	1.9%	3.4%	1.03 [0.57; 1.89]
Mehrafza M. (2019)[aa]	6	27	8	31	0.5%	1.1%	0.82 [0.24; 2.76]
Wang Z. (2019)	5	47	30	204	0.9%	1.6%	0.69 [0.25; 1.89]
Aghahoseini M. (2020)	1	38	8	24	0.9%	0.4%	0.05 [0.01; 0.47]
An J. (2020)	61	401	18	107	2.3%	3.6%	0.89 [0.50; 1.58]
Dong M. (2020)	41	90	122	370	2.4%	4.6%	1.70 [1.07; 2.72]
Qi Q. (2020)	34	147	249	1353	3.5%	5.3%	1.33 [0.89; 2.00]
Li M. (2021)	27	65	34	77	1.7%	3.0%	0.90 [0.46; 1.75]
Liu X. (2021)	73	387	71	363	5.6%	5.9%	0.96 [0.66; 1.38]
Luo L. (2021)	16	109	13	111	1.0%	2.3%	1.30 [0.59; 2.84]
Salama (2021)	0	32	6	37	0.5%	0.2%	0.07 [0.00; 1.38]
Xu J. (2021)	2	39	7	41	0.6%	0.6%	0.26 [0.05; 1.35]
Zheng Q. Z. (2021)	160	768	1104	6186	18.1%	8.4%	1.21 [1.01; 1.46]
Xia L. (2022)*	117	651	96	610	7.6%	6.8%	1.17 [0.87; 1.58]
Xia L. (2022)**	111	687	94	637	7.6%	6.8%	1.11 [0.83; 1.50]
Xia L. (2022)***	81	439	28	147	3.2%	4.5%	0.96 [0.60; 1.55]
Li L. (2022)	81	357	21	104	2.4%	3.9%	1.16 [0.68; 1.99]
Pan D. (2022)	31	142	25	68	2.5%	3.2%	0.48 [0.25; 0.91]
Wang Y. (2022)	25	223	143	837	5.0%	4.8%	0.61 [0.39; 0.96]
Gan R. X. (2022)	90	399	132	483	8.6%	6.6%	0.77 [0.57; 1.05]
Mo M. (2022)	12	72	30	116	1.8%	2.5%	0.57 [0.27; 1.21]
Total (fixed effect, 95% CI)		5347		12272	79.6%	--	1.01 [0.92; 1.12]
Total (random effects, 95% CI)					--	81.7%	0.93 [0.75; 1.15]
Heterogeneity: $Tau^2 = 0.1364$; $Chi^2 = 46.3$, df = 24 ($P < 0.01$); $I^2 = 48\%$							
**Subgroup = Daily**							
El-Toukhy T. (2004)	5	28	3	13	0.3%	0.7%	0.72 [0.14; 3.63]
Vijiver A. (2014)	27	47	90	137	1.8%	2.9%	0.70 [0.36; 1.39]
Kang J. (2018)	7	55	4	20	0.5%	0.9%	0.58 [0.15; 2.26]
Samsami A. (2018)	3	18	2	16	0.2%	0.5%	1.40 [0.20; 9.66]
Wageh A. (2018)	2	19	3	29	0.2%	0.5%	1.02 [0.15; 6.75]
Madani T. (2019)	11	44	8	35	0.6%	1.5%	1.12 [0.40; 3.19]
Mehrafza M. (2019)[a]	9	33	8	31	0.6%	1.3%	1.08 [0.35; 3.27]
Naserpoor L. (2020)	2	19	4	22	0.3%	0.5%	0.53 [0.09; 3.27]
Salemi S. (2021)	9	29	11	32	0.7%	1.4%	0.86 [0.29; 2.51]
Eleftheriadou (2022)	223	865	252	1121	15.2%	8.1%	1.20 [0.97; 1.47]
Total (fixed effect, 95% CI)		1157		1456	20.4%	--	1.10 [0.92; 1.33]
Total (random effects, 95% CI)					--	18.3%	1.10 [0.95; 1.28]
Heterogeneity: $Tau^2 = 0$; $Chi^2 = 4.3$, df = 9 ($P = 0.89$); $I^2 = 0\%$							
**Total (fixed effect, 95% CI)**		**6504**		**13728**	**100.0%**	**--**	**1.03 [0.95; 1.12]**
**Total (random effects, 95% CI)**					**--**	**100.0%**	**0.97 [0.84; 1.11]**

Heterogeneity: $Tau^2 = 0.0487$; $Chi^2 = 51.07$, df = 34 ($P = 0.03$); $I^2 = 33\%$
Test for subgroup differences (fixed effect): $Chi^2 = 0.65$, df = 1 ($P = 0.42$)
Test for subgroup differences (random effects): $Chi^2 = 2.02$, df = 1 ($P = 0.16$)

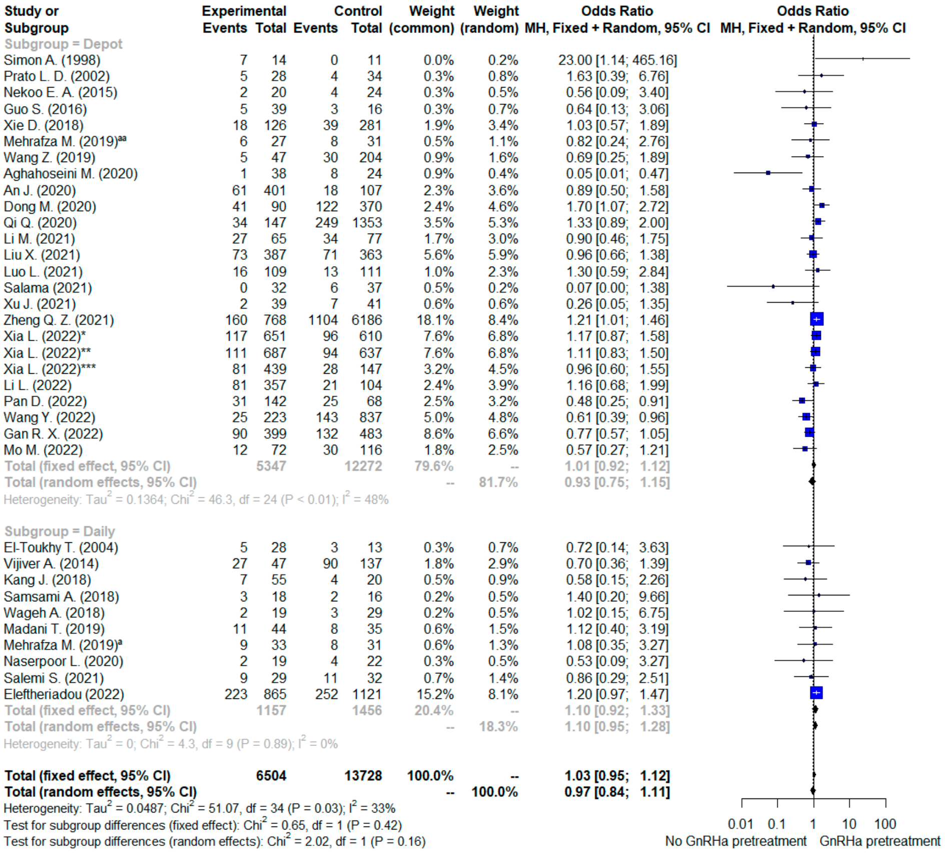

Supplementary Figure S4: (cont.)

(A) Clinical pregnancy rate

Study or Subgroup	Experimental Events	Experimental Total	Control Events	Control Total	Weight (common)	Weight (random)	Odds Ratio MH, Fixed + Random, 95% CI
**Subgroup = Within one cycle**							
Simon A. (1998)	14	53	11	52	0.2%	1.2%	1.34 [0.54; 3.30]
Prato L. D. (2002)	28	142	34	141	0.8%	2.1%	0.77 [0.44; 1.36]
El-Toukhy T. (2004)	28	117	13	117	0.3%	1.6%	2.52 [1.23; 5.15]
Davar (2007)	3	30	2	30	0.1%	0.4%	1.56 [0.24; 10.05]
Vijiver A. (2014)	47	280	137	849	1.7%	3.0%	1.05 [0.73; 1.51]
Nekoo E. A. (2015)	20	93	24	83	0.6%	1.7%	0.67 [0.34; 1.34]
Hebisha S. (2016)	72	110	42	100	0.5%	2.1%	2.62 [1.50; 4.57]
Guo S. (2016)	39	76	16	44	0.3%	1.5%	1.84 [0.86; 3.95]
Kang J. (2018)	55	113	20	49	0.4%	1.7%	1.38 [0.70; 2.71]
Movahedi. S. (2018)	9	60	7	40	0.2%	0.9%	0.83 [0.28; 2.45]
Samsami A. (2018)	18	109	16	107	0.4%	1.6%	1.12 [0.54; 2.34]
Wageh A. (2018)	19	37	29	58	0.3%	1.4%	1.06 [0.46; 2.41]
Madani T. (2019)	44	121	35	113	0.7%	2.2%	1.27 [0.74; 2.19]
Mehrafza M. (2019)[a]	33	100	31	103	0.6%	2.0%	1.14 [0.63; 2.07]
Mehrafza M. (2019)[aa]	27	93	31	103	0.6%	1.9%	0.95 [0.51; 1.76]
Wang Z. (2019)	47	92	204	396	1.1%	2.6%	0.98 [0.62; 1.55]
An J. (2020)	119	323	107	338	2.0%	3.1%	1.26 [0.91; 1.74]
Davar R. (2020)	8	31	6	31	0.1%	0.8%	1.45 [0.44; 4.81]
Dong M. (2020)	90	268	370	996	3.2%	3.3%	0.86 [0.64; 1.14]
Guerrero-Vargas J. J. (2020)	17	64	8	35	0.2%	1.1%	1.22 [0.47; 3.20]
Naserpoor L. (2020)	19	74	22	74	0.5%	1.6%	0.82 [0.40; 1.68]
Qi Q. (2020)	147	303	1353	2936	3.9%	3.5%	1.10 [0.87; 1.40]
Liu X. (2021)	387	514	363	514	2.7%	3.3%	1.27 [0.96; 1.67]
Luo L. (2021)	109	172	111	171	1.2%	2.6%	0.94 [0.60; 1.45]
Salama (2021)	32	70	37	70	0.6%	1.8%	0.75 [0.39; 1.46]
Salemi S. (2021)	29	93	32	95	0.7%	2.0%	0.89 [0.48; 1.64]
Siristatidis C. (2021)	62	159	24	221	0.4%	2.2%	5.25 [3.09; 8.92]
Xu J. (2021)	39	65	41	68	0.5%	1.7%	0.99 [0.49; 1.98]
Zheng Q. Z. (2021)	768	1518	6186	11456	21.6%	4.0%	0.87 [0.78; 0.97]
Eleftheriadou (2022)	865	1949	1121	2658	16.0%	3.9%	1.09 [0.97; 1.23]
Xia L. (2022)*	651	1165	610	1165	8.1%	3.8%	1.15 [0.98; 1.36]
Xia L. (2022)**	687	1133	637	1133	7.6%	3.8%	1.20 [1.01; 1.42]
Xia L. (2022)***	439	785	147	302	2.8%	3.4%	1.34 [1.03; 1.75]
Pan D. (2022)	142	290	68	194	1.3%	2.9%	1.78 [1.22; 2.58]
Wang Y. (2022)	223	309	837	1207	2.9%	3.3%	1.15 [0.87; 1.51]
Gan R. X. (2022)	399	846	483	1007	7.1%	3.7%	0.97 [0.81; 1.16]
Mo M. (2022)	72	155	116	294	1.3%	2.8%	1.33 [0.90; 1.97]
**Total (common effect, 95% CI)**		**11912**		**27350**	**93.6%**	.	**1.09 [1.04; 1.14]**
**Total (random effect, 95% CI)**					.	**86.8%**	**1.17 [1.04; 1.32]**
Heterogeneity: $Tau^2 = 0.0540$; $Chi^2 = 93.01$, df = 36 ($P < 0.01$); $I^2 = 61\%$							
**Subgroup = More than one cycle**							
Niu Z. (2013)	100	194	36	145	0.6%	2.5%	3.22 [2.01; 5.16]
Tsai H. W. (2017)	21	29	18	31	0.1%	0.9%	1.90 [0.64; 5.60]
Aghahoseini M. (2020)	38	88	24	90	0.4%	1.9%	2.09 [1.11; 3.92]
An J. (2020)	282	652	107	338	2.4%	3.3%	1.65 [1.25; 2.17]
Li L. (2022)	357	813	104	276	2.6%	3.3%	1.29 [0.98; 1.71]
Liu Y. (2022)	23	43	12	54	0.1%	1.3%	4.03 [1.67; 9.68]
**Total (common effect, 95% CI)**		**1819**		**934**	**6.4%**	.	**1.74 [1.47; 2.06]**
**Total (random effect, 95% CI)**					.	**13.2%**	**2.00 [1.29; 3.10]**
Heterogeneity: $Tau^2 = 0.0856$; $Chi^2 = 14.87$, df = 5 ($P = 0.01$); $I^2 = 66\%$							
**Total (common effect, 95% CI)**		**13731**		**28284**	**100.0%**	.	**1.13 [1.08; 1.18]**
**Total (random effect, 95% CI)**					.	**100.0%**	**1.27 [1.11; 1.44]**

Heterogeneity: $Tau^2 = 0.0895$; $Chi^2 = 135.24$, df = 42 ($P < 0.01$); $I^2 = 69\%$
Test for subgroup differences (common effect): $Chi^2 = 27.65$, df = 1 ($P < 0.01$)
Test for subgroup differences (random effects): $Chi^2 = 8.63$, df = 1 ($P < 0.01$)

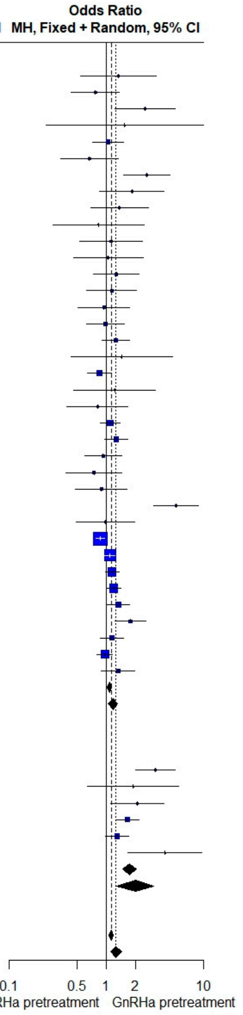

Supplementary Figure S5: Forest plots of meta-analysis for pregnancy outcomes following AC-FET cycles with and without GnRHa pretreatment: subgroups of GnRHa administration duration. **(A)** Clinical Pregnancy Rate. **(B)** Implantation Rate. **(C)** Live Birth Rate **(D)** Miscarriage Rate. *, **, and ***: three populations of infertile women were reported in the same study with matched or non-matched designs. a and aa: two different protocols were applied in the same study.

(B) Implantation rate

Study or Subgroup	Experimental Events	Experimental Total	Control Events	Control Total	Weight (common)	Weight (random)	Odds Ratio MH, Fixed + Random, 95% CI
Subgroup = Within one cycle							
Simon A. (1998)	17	179	15	167	0.5%	2.2%	1.06 [0.51; 2.20]
Prato L. D. (2002)	31	299	36	301	1.2%	3.3%	0.85 [0.51; 1.42]
El-Toukhy T. (2004)	43	269	26	258	0.8%	3.3%	1.70 [1.01; 2.86]
Davar (2007)	4	64	3	65	0.1%	0.7%	1.38 [0.30; 6.42]
Vijiver A. (2014)	47	239	137	733	2.0%	4.2%	1.06 [0.74; 1.54]
Nekoo E. A. (2015)	23	93	30	83	0.9%	2.6%	0.58 [0.30; 1.11]
Hebisha S. (2016)	142	322	60	285	1.3%	4.3%	2.96 [2.06; 4.24]
Guo S. (2016)	49	145	18	72	0.6%	2.6%	1.53 [0.81; 2.89]
Kang J. (2018)	76	181	24	88	0.7%	3.1%	1.93 [1.11; 3.36]
Movahedi. S. (2018)	9	136	10	98	0.4%	1.6%	0.62 [0.24; 1.60]
Madani T. (2019)	58	302	54	280	1.6%	3.9%	0.99 [0.66; 1.50]
Mehrafza M. (2019)[a]	34	245	37	258	1.1%	3.4%	0.96 [0.58; 1.59]
Mehrafza M. (2019)[aa]	35	235	37	258	1.1%	3.4%	1.05 [0.63; 1.72]
An J. (2020)	176	911	139	950	4.0%	5.1%	1.40 [1.09; 1.78]
Davar R. (2020)	8	52	6	57	0.2%	1.2%	1.55 [0.50; 4.80]
Luo L. (2021)	145	245	127	214	2.0%	4.2%	0.99 [0.68; 1.44]
Salama (2021)	54	117	65	127	1.2%	3.4%	0.82 [0.49; 1.35]
Salemi S. (2021)	54	198	48	201	1.3%	3.7%	1.20 [0.76; 1.88]
Xu J. (2021)	60	126	58	128	1.1%	3.4%	1.10 [0.67; 1.80]
Eleftheriadou (2022)	1040	2261	1322	3004	22.2%	5.9%	1.08 [0.97; 1.21]
Xia L. (2022)*	830	1994	743	1968	15.8%	5.8%	1.18 [1.03; 1.34]
Xia L. (2022)**	879	1879	830	1889	16.0%	5.8%	1.12 [0.99; 1.28]
Xia L. (2022)***	560	1309	190	491	5.7%	5.4%	1.18 [0.96; 1.46]
Gan R. X. (2022)	461	1213	549	1400	11.5%	5.7%	0.95 [0.81; 1.11]
Total (common effect, 95% CI)		13014		13375	93.2%	.	1.13 [1.07; 1.19]
Total (random effect, 95% CI)					.	88.2%	1.16 [1.02; 1.32]
Heterogeneity: $Tau^2 = 0.0421$; $Chi^2 = 53.17$, df = 23 (P < 0.01); $I^2 = 57\%$							
Subgroup = More than one cycle							
Niu Z. (2013)	127	390	45	280	1.3%	4.1%	2.52 [1.72; 3.70]
An J. (2020)	409	1849	139	950	5.2%	5.4%	1.66 [1.34; 2.05]
Liu Y. (2022)	29	86	15	107	0.3%	2.3%	3.12 [1.54; 6.32]
Total (common effect, 95% CI)		2325		1337	6.8%	.	1.89 [1.58; 2.26]
Total (random effect, 95% CI)					.	11.8%	2.07 [0.97; 4.43]
Heterogeneity: $Tau^2 = 0.0286$; $Chi^2 = 5.61$, df = 2 (P = 0.06); $I^2 = 64\%$							
**Total (common effect, 95% CI)**		**15339**		**14712**	**100.0%**	.	**1.18 [1.13; 1.25]**
**Total (random effect, 95% CI)**					.	**100.0%**	**1.24 [1.07; 1.45]**

Heterogeneity: $Tau^2 = 0.0793$; $Chi^2 = 88.00$, df = 26 (P < 0.01); $I^2 = 70\%$
Test for subgroup differences (common effect): $Chi^2 = 28.91$, df = 1 (P < 0.01)
Test for subgroup differences (random effects): $Chi^2 = 9.64$, df = 1 (P < 0.01)

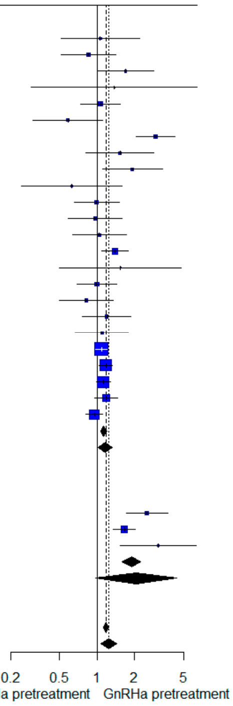

(C) Live birth rate

Study or Subgroup	Experimental Events	Experimental Total	Control Events	Control Total	Weight (common)	Weight (random)	Odds Ratio MH, Fixed + Random, 95% CI
Subgroup = Within one cycle							
El-Toukhy T. (2004)	23	117	10	117	0.3%	2.3%	2.62 [1.19; 5.78]
Vijiver A. (2014)	41	275	127	839	1.8%	4.1%	0.98 [0.67; 1.44]
Guo S. (2016)	34	76	11	44	0.3%	2.2%	2.43 [1.07; 5.51]
Kang J. (2018)	47	113	16	49	0.4%	2.6%	1.47 [0.73; 2.97]
Madani T. (2019)	38	121	31	113	0.7%	3.2%	1.21 [0.69; 2.13]
Wang Z. (2019)	37	92	126	396	1.0%	3.7%	1.44 [0.90; 2.30]
An J. (2020)	90	323	76	338	1.8%	4.2%	1.33 [0.94; 1.89]
Dong M. (2020)	47	268	240	996	2.8%	4.2%	0.67 [0.47; 0.95]
Guerrero-Vargas J. J. (2020)	11	64	3	35	0.1%	1.1%	2.21 [0.57; 8.54]
Qi Q. (2020)	106	303	1051	2936	4.3%	4.7%	0.97 [0.75; 1.24]
Liu X. (2021)	309	514	288	514	3.9%	4.7%	1.18 [0.92; 1.52]
Luo L. (2021)	85	172	92	171	1.6%	3.9%	0.84 [0.55; 1.28]
Salemi S. (2021)	20	93	21	95	0.6%	2.7%	0.97 [0.48; 1.93]
Siristatidis C. (2021)	58	159	9	221	0.2%	2.5%	13.53 [6.45; 28.38]
Xu J. (2021)	31	65	32	68	0.6%	2.7%	1.03 [0.52; 2.03]
Zheng Q. Z. (2021)	578	1518	4750	11456	23.3%	5.1%	0.87 [0.78; 0.97]
Eleftheriadou (2022)	716	1949	978	2658	17.7%	5.1%	1.00 [0.88; 1.13]
Xia L. (2022)*	527	1165	498	1165	9.2%	5.0%	1.11 [0.94; 1.30]
Xia L. (2022)**	570	1133	535	1133	9.0%	5.0%	1.13 [0.96; 1.33]
Xia L. (2022)***	357	785	117	302	3.1%	4.6%	1.32 [1.01; 1.73]
Pan D. (2022)	106	290	43	194	1.1%	3.9%	2.02 [1.34; 3.06]
Wang Y. (2022)	195	309	685	1207	3.5%	4.7%	1.30 [1.01; 1.69]
Gan R. X. (2022)	302	846	343	1007	6.8%	4.9%	1.07 [0.89; 1.30]
Mo M. (2022)	57	155	73	294	1.1%	3.9%	1.76 [1.16; 2.68]
Total (common effect, 95% CI)		10905		26348	95.2%	.	1.08 [1.02; 1.13]
Total (random effect, 95% CI)					.	91.1%	1.28 [1.03; 1.60]
Heterogeneity: $Tau^2 = 0.1521$; $Chi^2 = 103.51$, df = 23 (P < 0.01); $I^2 = 78\%$							
Subgroup = More than one cycle							
An J. (2020)	209	652	76	338	2.3%	4.4%	1.63 [1.20; 2.20]
Li L. (2022)	246	813	72	276	2.5%	4.4%	1.23 [0.90; 1.67]
Total (common effect, 95% CI)		1465		614	4.8%	.	1.42 [1.14; 1.76]
Total (random effect, 95% CI)					.	8.9%	1.42 [0.24; 8.39]
Heterogeneity: $Tau^2 = < 0.0001$; $Chi^2 = 1.61$, df = 1 (P = 0.20); $I^2 = 38\%$							
**Total (common effect, 95% CI)**		**12370**		**26962**	**100.0%**	.	**1.09 [1.04; 1.15]**
**Total (random effect, 95% CI)**					.	**100.0%**	**1.29 [1.06; 1.57]**

Heterogeneity: $Tau^2 = 0.1290$; $Chi^2 = 111.31$, df = 25 (P < 0.01); $I^2 = 78\%$
Test for subgroup differences (common effect): $Chi^2 = 5.93$, df = 1 (P = 0.01)
Test for subgroup differences (random effects): $Chi^2 = 0.31$, df = 1 (P = 0.58)

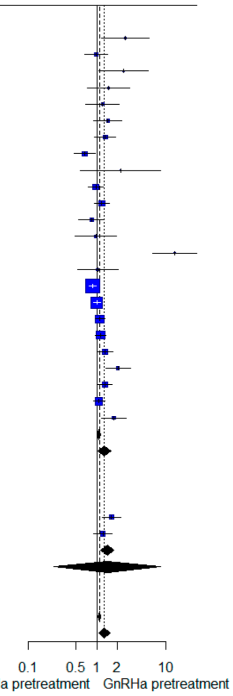

Supplementary Figure S5: (cont.)

(D) Miscarriage rate

Study or Subgroup	Experimental Events	Experimental Total	Control Events	Control Total	Weight (common)	Weight (random)	Odds Ratio MH, Fixed + Random, 95% CI
**Subgroup = Within one cycle**							
Simon A. (1998)	7	14	0	11	0.0%	0.5%	23.00 [1.14; 465.16]
Prato L. D. (2002)	5	28	4	34	0.3%	1.7%	1.63 [0.39; 6.76]
El-Toukhy T. (2004)	5	28	3	13	0.3%	1.5%	0.72 [0.14; 3.63]
Vijiver A. (2014)	27	47	90	137	1.8%	3.6%	0.70 [0.36; 1.39]
Nekoo E. A. (2015)	2	20	4	24	0.3%	1.2%	0.56 [0.09; 3.40]
Guo S. (2016)	5	39	3	16	0.3%	1.5%	0.64 [0.13; 3.06]
Kang J. (2018)	7	55	4	20	0.5%	1.9%	0.58 [0.15; 2.26]
Samsami A. (2018)	3	18	2	16	0.2%	1.1%	1.40 [0.20; 9.66]
Wageh A. (2018)	2	19	3	29	0.2%	1.1%	1.02 [0.15; 6.75]
Madani T. (2019)	11	44	8	35	0.6%	2.5%	1.12 [0.40; 3.19]
Mehrafza M. (2019)[a]	9	33	8	31	0.6%	2.3%	1.08 [0.35; 3.27]
Mehrafza M. (2019)[aa]	6	27	8	31	0.5%	2.1%	0.82 [0.24; 2.76]
Wang Z. (2019)	5	47	30	204	0.9%	2.6%	0.69 [0.25; 1.89]
An J. (2020)	17	119	18	107	1.5%	3.4%	0.82 [0.40; 1.70]
Dong M. (2020)	41	90	122	370	2.4%	4.3%	1.70 [1.07; 2.72]
Naserpoor L. (2020)	2	19	4	22	0.3%	1.2%	0.53 [0.09; 3.27]
Qi Q. (2020)	34	147	249	1353	3.5%	4.5%	1.33 [0.89; 2.00]
Liu X. (2021)	73	387	71	363	5.6%	4.6%	0.96 [0.66; 1.38]
Luo L. (2021)	16	109	13	111	1.0%	3.2%	1.30 [0.59; 2.84]
Salama (2021)	0	32	6	37	0.5%	0.6%	0.07 [0.00; 1.38]
Salemi S. (2021)	9	29	11	32	0.7%	2.4%	0.86 [0.29; 2.51]
Siristatidis C. (2021)	4	62	15	24	1.9%	1.9%	0.04 [0.01; 0.15]
Xu J. (2021)	2	39	7	41	0.6%	1.4%	0.26 [0.05; 1.35]
Zheng Q. Z. (2021)	160	768	1104	6186	18.1%	5.0%	1.21 [1.01; 1.46]
Eleftheriadou (2022)	223	865	252	1121	15.3%	5.0%	1.20 [0.97; 1.47]
Xia L. (2022)*	117	651	96	610	7.6%	4.8%	1.17 [0.87; 1.58]
Xia L. (2022)**	111	687	94	637	7.7%	4.8%	1.11 [0.83; 1.50]
Xia L. (2022)***	81	439	28	147	3.2%	4.2%	0.96 [0.60; 1.55]
Pan D. (2022)	31	142	25	68	2.5%	3.7%	0.48 [0.25; 0.91]
Wang Y. (2022)	25	223	143	837	5.0%	4.3%	0.61 [0.39; 0.96]
Gan R. X. (2022)	90	399	132	483	8.7%	4.8%	0.77 [0.57; 1.05]
Mo M. (2022)	12	72	30	116	1.8%	3.4%	0.57 [0.27; 1.21]
Total (common effect, 95% CI)		5698		13266	94.7%	.	1.02 [0.94; 1.11]
Total (random effect, 95% CI)					.	91.2%	0.87 [0.69; 1.09]
Heterogeneity: $Tau^2 = 0.2118$; $Chi^2 = 66.99$, df = 31 ($P < 0.01$); $I^2 = 54\%$							
**Subgroup = More than one cycle**							
Aghahoseini M. (2020)	1	38	8	24	0.9%	0.9%	0.05 [0.01; 0.47]
An J. (2020)	44	282	18	107	2.1%	3.8%	0.91 [0.50; 1.67]
Li L. (2022)	81	357	21	104	2.4%	4.0%	1.16 [0.68; 1.99]
Total (common effect, 95% CI)		677		235	5.3%	.	0.88 [0.60; 1.28]
Total (random effect, 95% CI)					.	8.8%	0.50 [0.01; 24.11]
Heterogeneity: $Tau^2 = 2.0772$; $Chi^2 = 7.31$, df = 2 ($P = 0.03$); $I^2 = 73\%$							
**Total (common effect, 95% CI)**		**6375**		**13501**	**100.0%**	.	**1.01 [0.93; 1.10]**
**Total (random effect, 95% CI)**					.	**100.0%**	**0.85 [0.67; 1.07]**

Heterogeneity: $Tau^2 = 0.2630$; $Chi^2 = 74.49$, df = 34 ($P < 0.01$); $I^2 = 54\%$
Test for subgroup differences (common effect): $Chi^2 = 0.59$, df = 1 ($P = 0.44$)
Test for subgroup differences (random effects): $Chi^2 = 0.37$, df = 1 ($P = 0.54$)

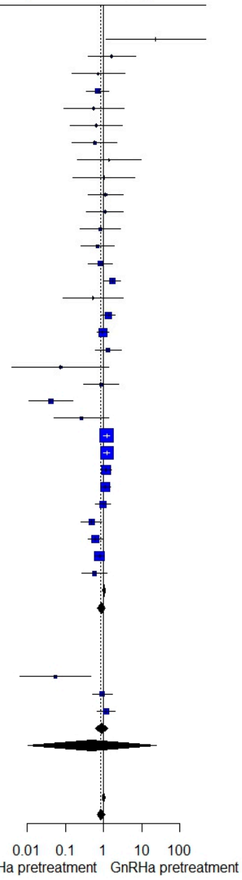

Supplementary Figure S5: (cont.)

(A) Clinical pregnancy rate

Study	Experimental Events	Experimental Total	Control Events	Control Total	Weight (common)	Weight (random)	Odds Ratio MH, Fixed + Random, 95% CI
Tsai H. W. (2017)	21	29	18	31	1.6%	2.0%	1.90 [0.64; 5.60]
Wageh A. (2018)	19	37	29	58	3.6%	3.4%	1.06 [0.46; 2.41]
Xie D. (2018)	33	48	39	98	2.6%	4.3%	3.33 [1.60; 6.92]
Mehrafza M. (2019)[a]	11	26	5	16	1.2%	1.3%	1.61 [0.43; 5.99]
Mehrafza M. (2019)[aa]	4	12	5	16	0.9%	0.9%	1.10 [0.22; 5.45]
Aghahoseini M. (2020)	38	88	24	90	4.4%	5.8%	2.09 [1.11; 3.92]
Qi Q. (2020)	16	32	182	354	4.9%	4.4%	0.95 [0.46; 1.95]
Liu X. (2021)	387	514	363	514	29.3%	30.1%	1.27 [0.96; 1.67]
Luo L. (2021)	109	172	111	171	13.3%	11.8%	0.94 [0.60; 1.45]
Salemi S. (2021)	29	93	32	95	7.1%	6.1%	0.89 [0.48; 1.64]
Wang Y. (2022)	223	309	837	1207	31.0%	29.9%	1.15 [0.87; 1.51]
**Total (fixed effect, 95% CI)**		**1360**		**2650**	**100.0%**	**--**	**1.24 [1.06; 1.44]**
**Total (random effects, 95% CI)**					**--**	**100.0%**	**1.23 [1.01; 1.51]**

Heterogeneity: $Tau^2 = 0$; $Chi^2 = 14.10$, df = 10 (P = 0.17); $I^2 = 29\%$

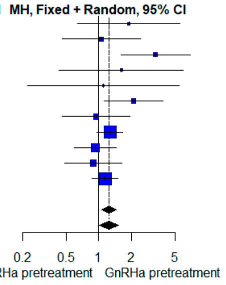

(B) Implantation rate

Study	Experimental Events	Experimental Total	Control Events	Control Total	Weight (common)	Weight (random)	Odds Ratio MH, Fixed + Random, 95% CI
Xie D. (2018)	41	102	50	221	17.3%	30.8%	2.30 [1.39; 3.81]
Luo L. (2021)	145	245	127	214	50.8%	36.2%	0.99 [0.68; 1.44]
Salemi S. (2021)	54	198	48	201	31.8%	33.0%	1.20 [0.76; 1.88]
**Total (fixed effect, 95% CI)**		**545**		**636**	**100.0%**	**--**	**1.28 [1.00; 1.65]**
**Total (random effects, 95% CI)**					**--**	**100.0%**	**1.37 [0.46; 4.03]**

Heterogeneity: $Tau^2 = 0.1380$; $Chi^2 = 7.00$, df = 2 (P = 0.03); $I^2 = 71\%$

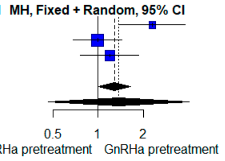

(C) Live birth rate

Study	Experimental Events	Experimental Total	Control Events	Control Total	Weight (common)	Weight (random)	Odds Ratio MH, Fixed + Random, 95% CI
Xie D. (2018)	27	48	30	98	2.9%	11.2%	2.91 [1.43; 5.95]
Qi Q. (2020)	14	32	122	354	3.8%	10.8%	1.48 [0.71; 3.08]
Liu X. (2021)	309	514	288	514	38.2%	24.1%	1.18 [0.92; 1.52]
Luo L. (2021)	85	172	92	171	15.5%	18.5%	0.84 [0.55; 1.28]
Salemi S. (2021)	20	93	21	95	5.4%	11.6%	0.97 [0.48; 1.93]
Wang Y. (2022)	195	309	685	1207	34.2%	23.8%	1.30 [1.01; 1.69]
**Total (fixed effect, 95% CI)**		**1168**		**2439**	**100.0%**	**--**	**1.22 [1.05; 1.42]**
**Total (random effects, 95% CI)**					**--**	**100.0%**	**1.26 [0.84; 1.88]**

Heterogeneity: $Tau^2 = 0.0846$; $Chi^2 = 9.73$, df = 5 (P = 0.08); $I^2 = 49\%$

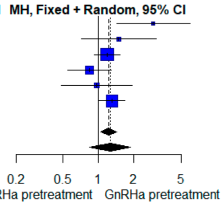

(D) Miscarriage rate

Study	Experimental Events	Experimental Total	Control Events	Control Total	Weight (common)	Weight (random)	Odds Ratio MH, Fixed + Random, 95% CI
Wageh A. (2018)	2	19	3	29	1.3%	6.6%	1.02 [0.15; 6.75]
Xie D. (2018)	5	33	9	39	4.4%	11.5%	0.60 [0.18; 1.99]
Aghahoseini M. (2020)	1	38	8	24	6.0%	5.5%	0.05 [0.01; 0.47]
Qi Q. (2020)	1	16	58	182	5.5%	5.9%	0.14 [0.02; 1.11]
Liu X. (2021)	73	387	71	363	37.5%	21.2%	0.96 [0.66; 1.38]
Luo L. (2021)	16	109	13	111	6.9%	16.2%	1.30 [0.59; 2.84]
Salemi S. (2021)	9	29	11	32	4.5%	12.9%	0.86 [0.29; 2.51]
Wang Y. (2022)	25	223	143	837	33.7%	20.2%	0.61 [0.39; 0.96]
**Total (fixed effect, 95% CI)**		**854**		**1617**	**100.0%**	**--**	**0.75 [0.59; 0.95]**
**Total (random effects, 95% CI)**					**--**	**100.0%**	**0.66 [0.33; 1.32]**

Heterogeneity: $Tau^2 = 0.3755$; $Chi^2 = 12.71$, df = 7 (P = 0.08); $I^2 = 45\%$

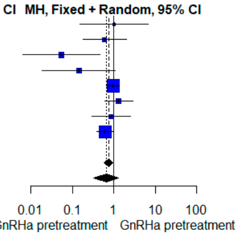

Supplementary Figure S6: Forest plots of meta-analysis for pregnancy outcomes following AC-FET cycles with and without GnRHa pretreatment in PCOS patients. **(A)** Clinical Pregnancy Rate. **(B)** Implantation Rate. **(C)** Live Birth Rate **(D)** Miscarriage Rate.

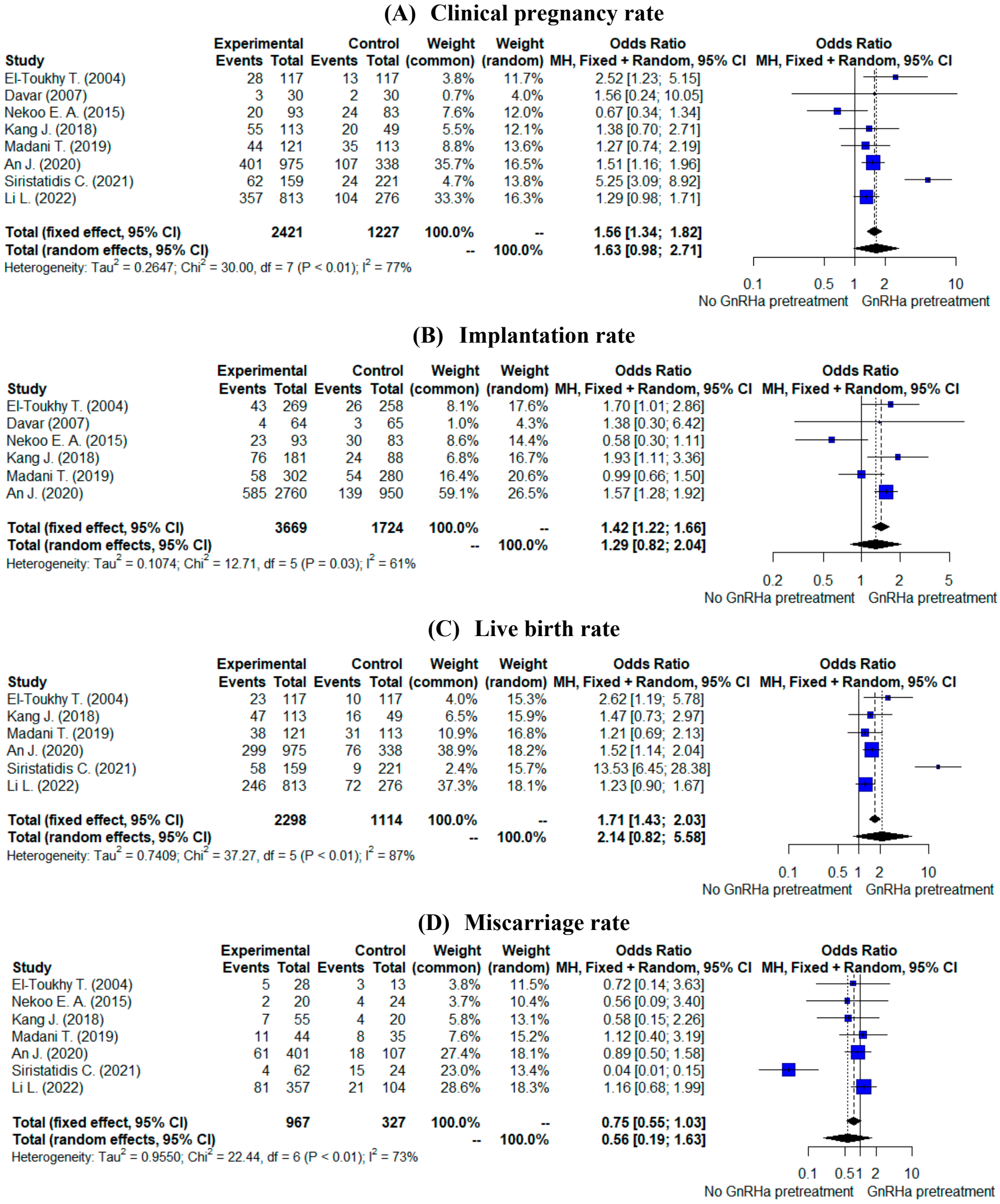

Supplementary Figure S7: Forest plots of meta-analysis for pregnancy outcomes following AC-FET cycles with and without GnRHa pretreatment in ovulatory women with regular cycles. **(A)** Clinical Pregnancy Rate. **(B)** Implantation Rate. **(C)** Live Birth Rate **(D)** Miscarriage Rate.
